# Supplementary material for: 2D MXenes polar catalysts for multi-renewable energy harvesting applications
Source: Nat Commun. 2023 Jul 13;14:4183. doi: 10.1038/s41467-023-39791-w (PMC10345010; doi:10.1038/s41467-023-39791-w)
Supplement: Supplementary file 1 — Supplementary Information [file 41467_2023_39791_MOESM1_ESM.pdf]

Electronic Supplementary Information for the Manuscript

Multiple renewable energy harvesting over 2D MXenes polar catalysts

Xiaoyang Pan,[1*] Xuhui Yang,[2] Maoqing Yu,[1,3] Xiaoxiao Lu,[1,2] Hao Kang,[2] Min-Quan Yang,[2*] Qingrong Qian,[2] Xiaojing Zhao,[1] Shijing Liang[3*], Zhenfeng Bian[4*]

[1]*College of Chemical Engneering and Materials, Quanzhou Normal University, Quanzhou, 362000, P. R. China.*

[2]*College of Environmental and Resource Sciences, College of Carbon Neutral Modern Industry, Fujian Key Laboratory of Pollution Control & Resource Reuse, Fujian Normal University, Fuzhou 350007, P. R. China*

[3]*National Engineering Research Center of Chemical Fertilizer Catalyst Fuzhou University, Fuzhou 350002 P. R. China*

[4]*Education Ministry Key and International Joint Lab of Resource Chemistry and Shanghai Key Lab of Rare Earth Functional Materials, Shanghai Normal University, Shanghai 200234, P. R. China.*

Corresponding Author: Prof. Xiaoyang Pan, xypan@qztc.edu.cn; Prof. Min-Quan Yang, yangmq@fjnu.edu.cn; Prof. Shijing Liang, sjliang2012@fzu.edu.cn; Prof. Zhenfeng Bian, bianzhenfeng@shnu.edu.cn

Supplementary Methods.

Synthesis.

Synthesis of CdS. 0.2665 g of cadmium acetate and 0.3045g thiourea were dissolved in 75 mL water. The resultant solution was stirred for 30 min and then hydrothermally heated at 433 K for 12h. After that, the resulting suspension was washed with deionized water three times and dried in an oven at 333 K.

Synthesis of BiOBr. Bi $(NO_3)_3$ (0.7955g) and KBr (0.1952 g) were poured into a mortar, and then ground for 15 min. Subsequently, the powder was washed with deionized water for three times, and dried at 333 K.

Synthesis of $BiVO_4$. 1mmol of Bi $(NO_3)_3$ was dissolved in 10 Ml of HNO_3 (4 M) solution. 1 mmol of NH_4VO_3 was added into 10 ml of 2 M NaOH solution. After stirring for 0.5 h, NH_4VO_3 solution was dropped into $Bi(NO_3)_3$ solution to form a yellow suspension. After stirring for 0.5 h, the pH of the obtained suspension was adjusted to 7 with 2 M NaOH solution. Subsequently, the suspension was stirred for 0.5 h, and then hydrothermally treated at 160℃ for 6 h. The product was washed with distilled water three times and then dried at 333 K for 4 hours.

Synthesis of Bi_2WO_6. 1 mmol of Na_2WO_4, 2 mmol of $Bi(NO_3)_3$ and 0.1 mmol KBr were dissolved in 80 ml of deionized water. Then the resultant solution was hydrothermally treated at 393 K for 24h. The product was washed with water for 3 times and then dried in an oven at 333 K.

Synthesis of C_3N_4. 50 g of urea was placed in a crucible, and then calcined at 773 K for 4 hours. The heating rate was 10 K per minute.

Synthesis of ZnO. 0.005 M of NaOH ethanol solution was slowly dropped into 0.005 M of Zn $(NO_3)_2$ ethanol solution. The mixed solution was hdrothermally treated

at 403 K for 12 hours. The product was washed with deionized water three times, and then dried at 333 K.

Theoretical calculation.

The calculation is carried out within the framework of density functional theory of VASP.[1] Perdew-Burke-Ernzerhof (PBE) is used to exchange correlation functional, and generalized gradient approximation (GGA) is used to deal with the exchange and correlation effects self-consistently.[2] The electron wave function is expanded in the plane wave basis group with the energy cut-off of 600 eV. For structural optimizations and static calculations, the convergences for total energy and force are set as 1×10^{-6} eV and 0.01 eV/Å respectively. Brillouin zone is sampled on a $21 \times 21 \times 1$ grid in the gamma center scheme. In order to prevent periodic interaction along the Z direction, a vacuum space of 20 Å is set between adjacent layers. In addition, the DFT-D3 method[3] is adopted to correct the VDW interactions. Since functional groups and strains broke the inversion symmetry of the initial structures of MXene, we call off the symmetry. The adsorption energy and Barder charge for O_2 adsorption is performed according to previous report.[4] In details, the models for monolayer and bilayer Ti_3C_2 were built by 3×3 supercells with a 20 Å vacuum layer along the *z*-axis. The adsorption energy E_{ads} was calculated using the following formula

$$E_{ads} = E_{total} - E_{\mathrm{substrate}} - E_{O_2}$$

where E_{total}, $E_{\mathrm{substrate}}$ and E_{O_2} stand for the total energy of the O_2 adsorbed to Ti_3C_2 slabs, Ti_3C_2 slabs and O_2 molecule.

Characterization.

PFM is based on the atomic force microscopy, with an AC drive voltage applied to the conductive tip. Conductive Pt/Ir-coated silicon probes (EFM, Nanoworld) were used with a nominal spring constant of ~2.8 nN/nm and a free-air resonance frequency of ~75 kHz. The typical drive frequency was in the range of 320 to 380 kHz, depending

on the contact resonant frequency. The Fourier transform infrared spectroscopy (FTIR) data were collected using a NICOLET IS10 (Thermo Fisher) spectrophotometer at a resolution of $4cm^{-1}$. ζ-potential measurements were recorded at room temperature on a dynamic light scattering analyzer (BI-200SM). Inductively coupled plasma optical emission spectrometry (ICP-OES) was performed on Ultima 2 to determine the leaching of Al in the solution.

Calculation of the concentrations of the $\bullet O_2^-$ and •OH radicals

The amounts of $\bullet O_2^-$ and •OH are determined by nitroblue tetrazolium (NBT) transformation and terephthalic acid (TA) photoluminescence probing means, respectively. NBT (Maximum absorption peak: ca. 260 nm) can react with $\bullet O_2^-$ in a molar ratio of 1:4, which can be used to calculate the quantities of $\bullet O_2^-$ over the sample. The $\bullet O_2^-$ production rate can be obtained by recording the concentration change of NBT. TA could react with •OH in a molar ratio of 1:1 to generate 2-hydroxyterephthalic acid (2-HA) with strong fluorescence at ca. 425 nm (excitation at 315 nm), The generation of •OH is quantified by monitoring the PL intensity of 2-HA with the excitation wavelength of 315 nm. The PL intensity is detected on a fluorescence spectrophotometer (HITACHI, F-7000).

Supplementary Equations:

$$\cdot O_2^- + H^+ \rightarrow HO_2^\cdot \quad (1)$$

$$2HO_2^\cdot \rightarrow H_2O_2 + O_2 \quad (2)$$

$$H_2O_2 + \cdot O_2^- \rightarrow \cdot OH + O_2 + OH^- \quad (3)$$

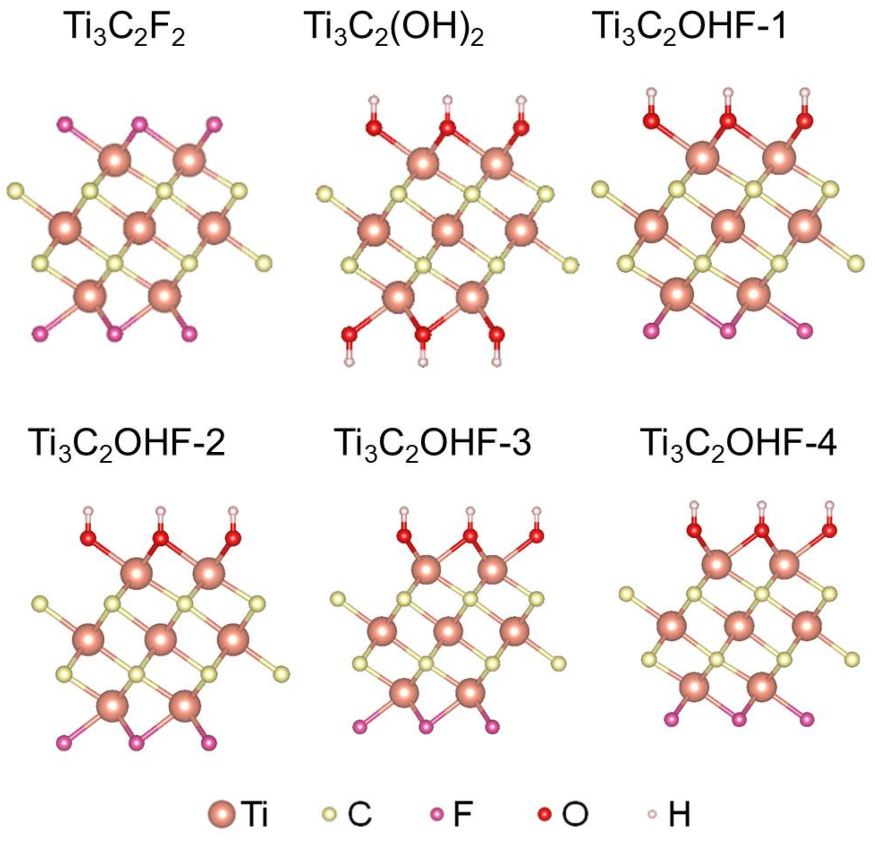

Supplementary Fig. 1 Crystal structures of $Ti_3C_2T_X$ monolayers.

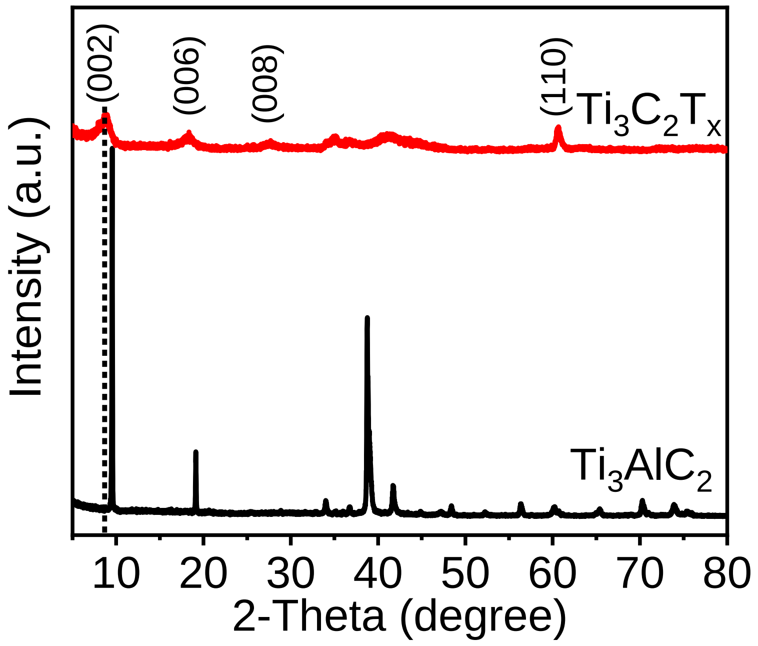

Supplementary Fig. 2 XRD patterns of the Ti_3AlC_2 and $Ti_3C_2T_X$.

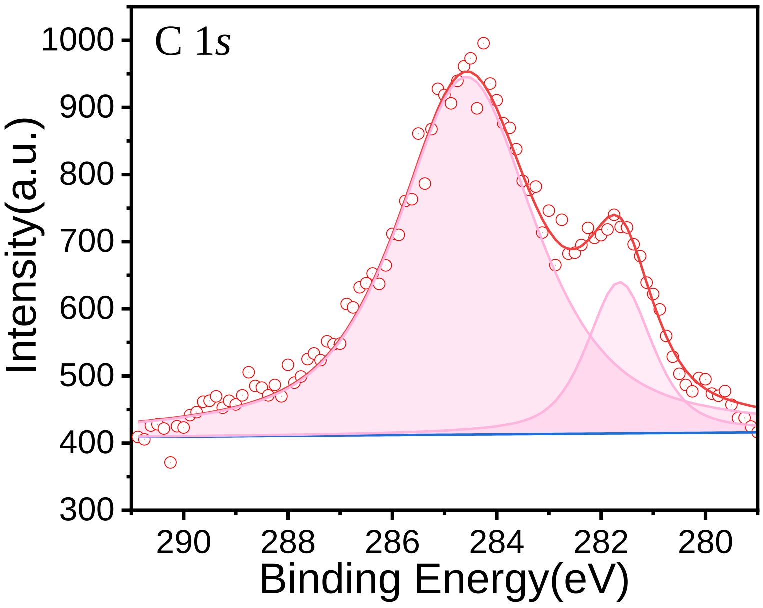

Supplementary Fig. 3 XPS spectrum of the C 1*s* of $Ti_3C_2T_X$.

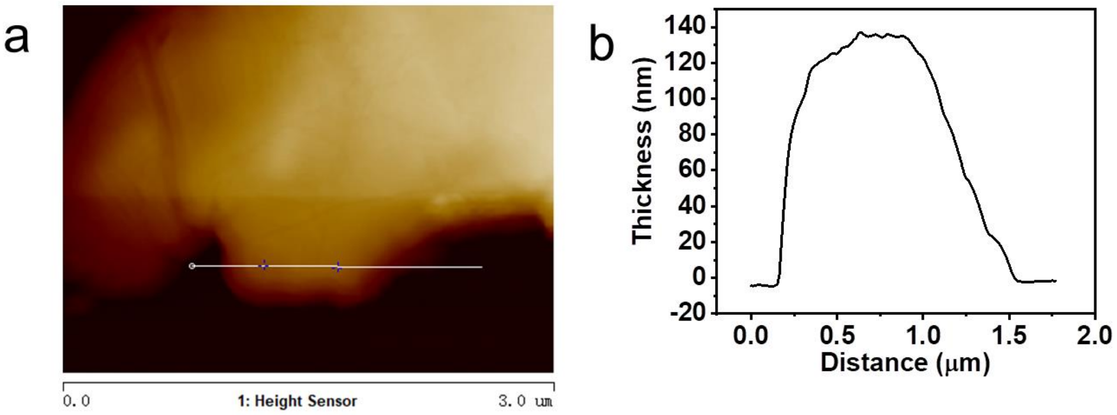

Supplementary Fig. 4 a AFM image and **b** thickness profile of $Ti_3C_2T_X$.

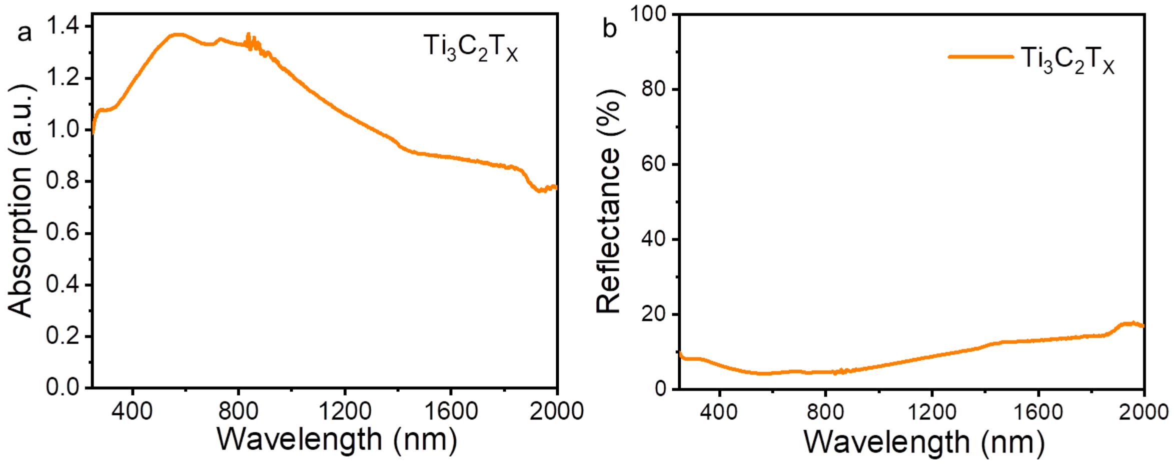

Supplementary Fig. 5 Optical property of the $Ti_3C_2T_X$. a Absorption and **b** reflectance spectra of the $Ti_3C_2T_X$.

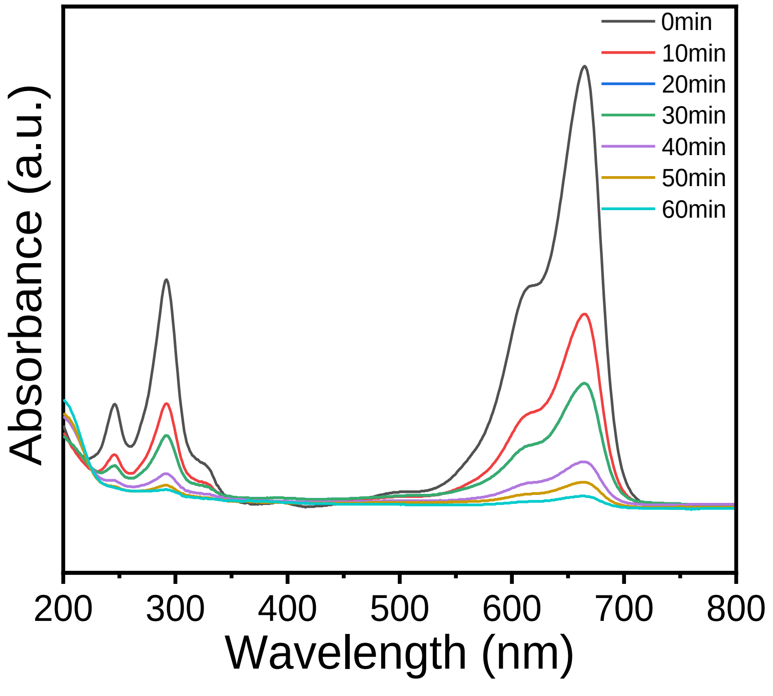

Supplementary Fig. 6 UV-vis absorption spectra of piezocatalytic degradation of MB solution with $Ti_3C_2T_X$ under different reaction time.

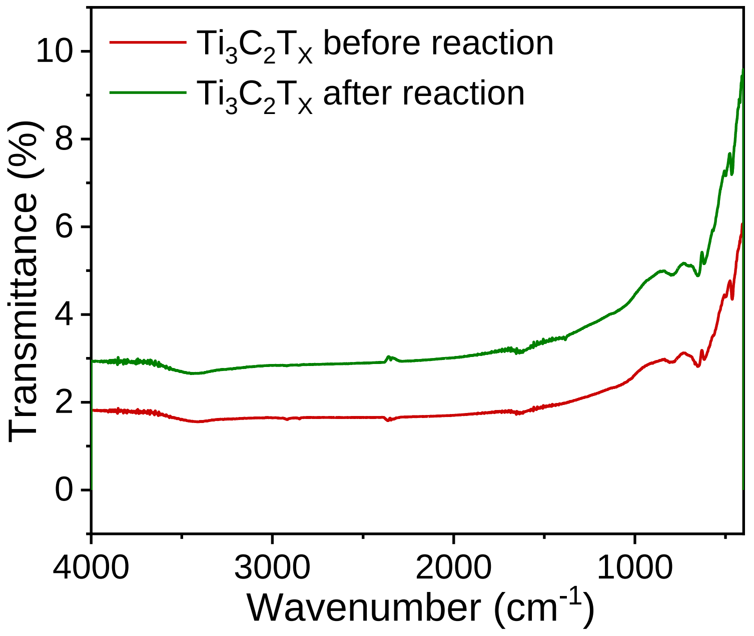

Supplementary Fig. 7 FTIR spectra of $Ti_3C_2T_X$ before and after reaction.

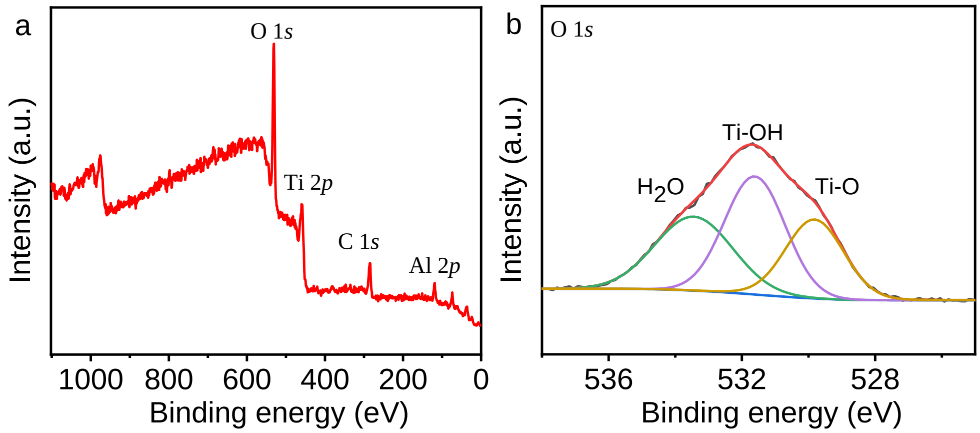

Supplementary Fig. 8 XPS spectra of Ti_3AlC_2 after sonication. a survey spectrum. **b** O 1s.

Supplementary Note 2: ICP analysis is used to determine the Al leaching in the solution. The result shows that 0.5 mg/L of Al could be detected in the reaction solution.

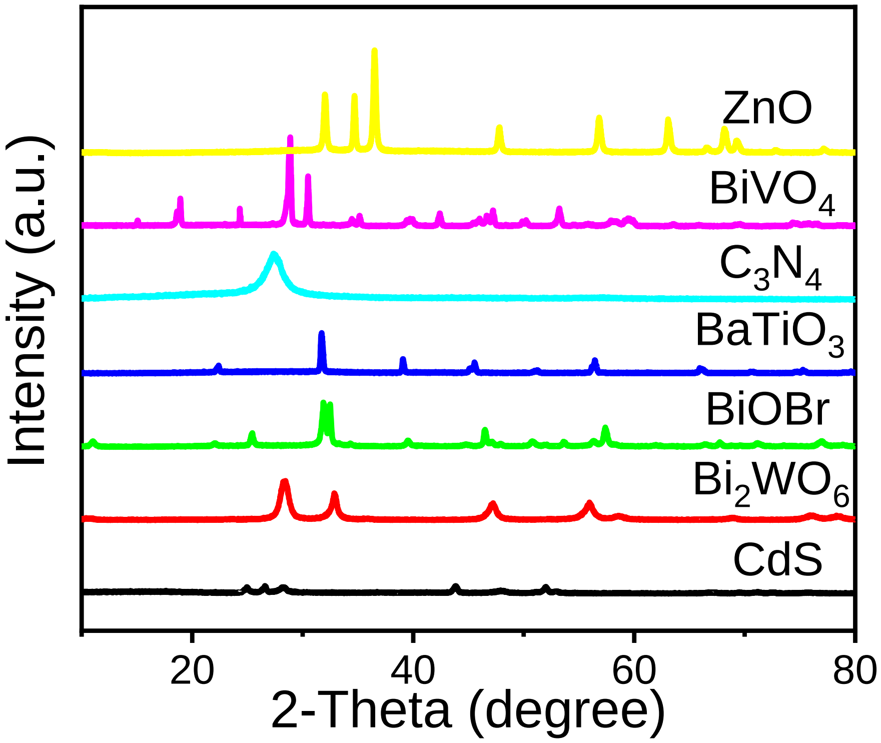

Supplementary Fig. 9 XRD patterns of the samples.

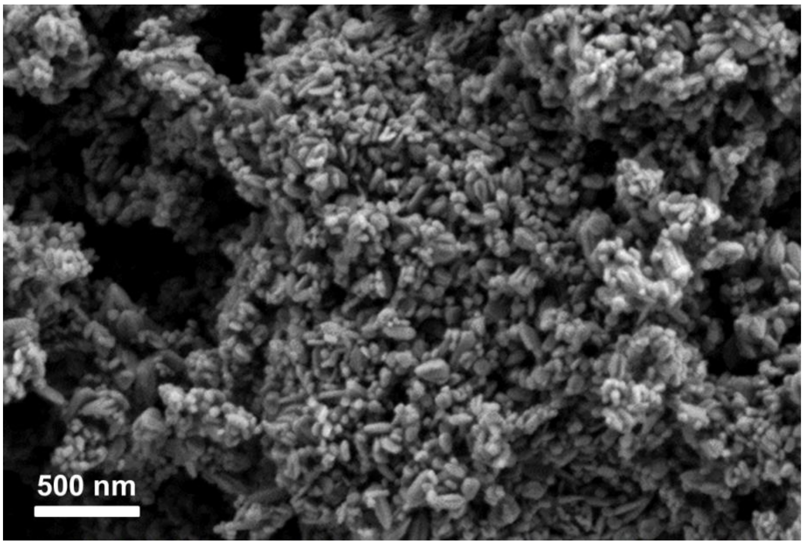

Supplementary Fig. 10 SEM image of ZnO.

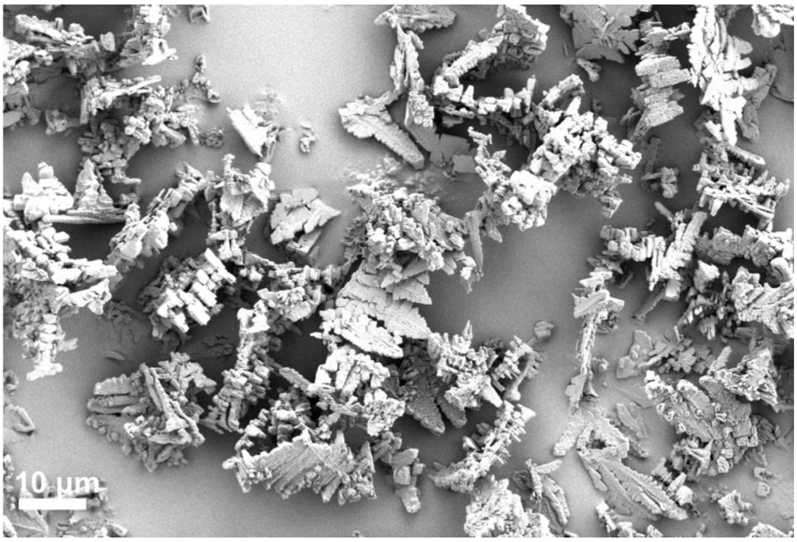

Supplementary Fig. 11 SEM image of $BiVO_4$.

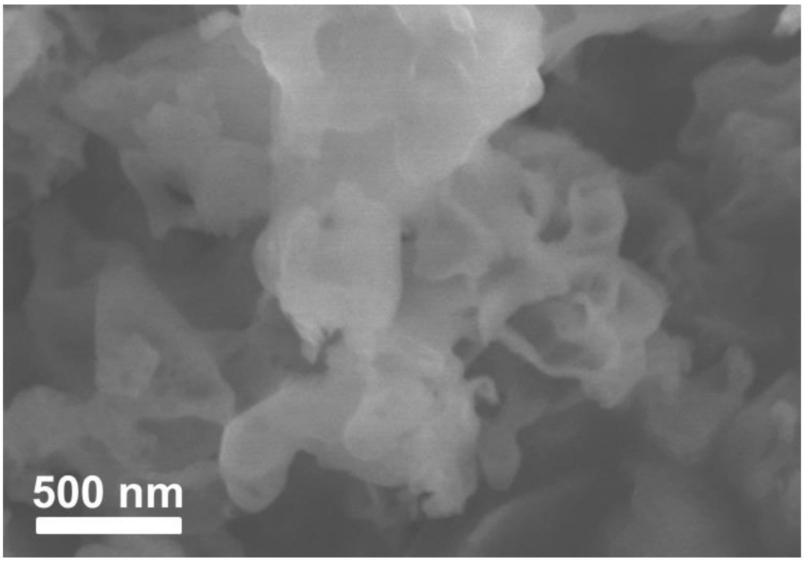

Supplementary Fig. 12 SEM image of C_3N_4.

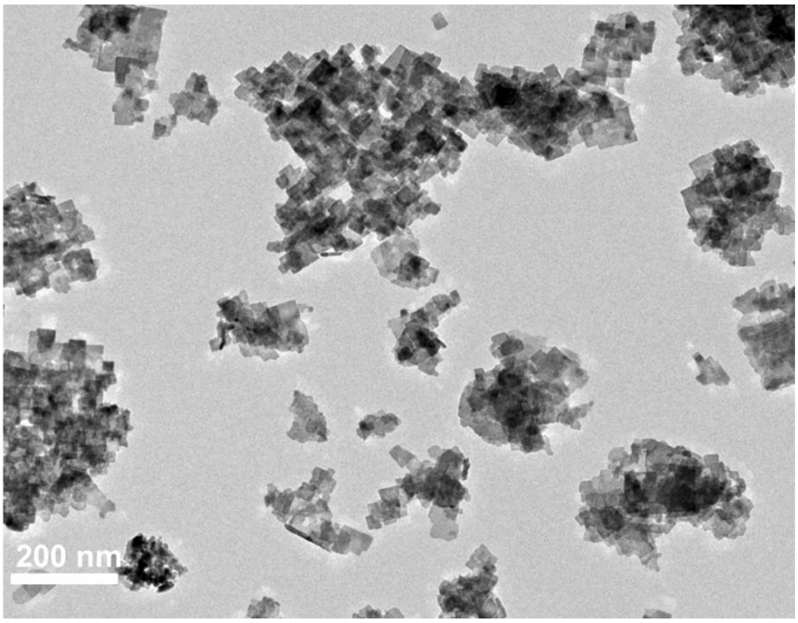

Supplementary Fig. 13 TEM image of Bi_2WO_6.

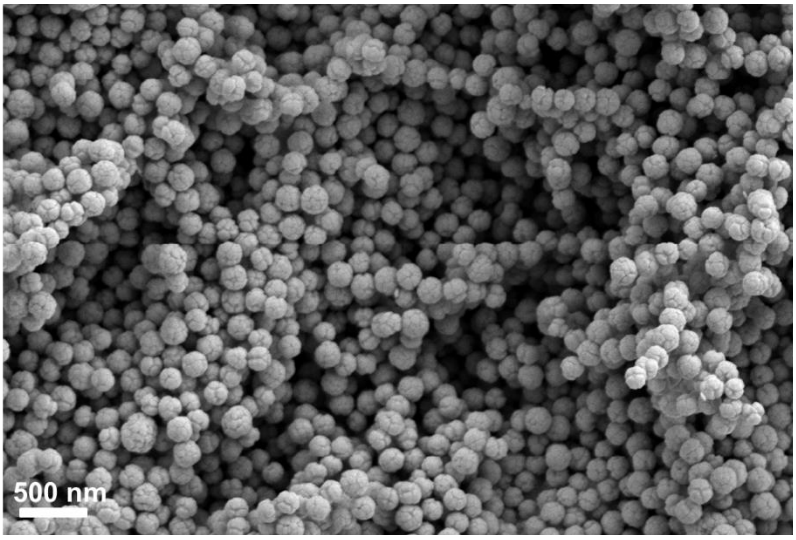

Supplementary Fig. 14 SEM image of CdS.

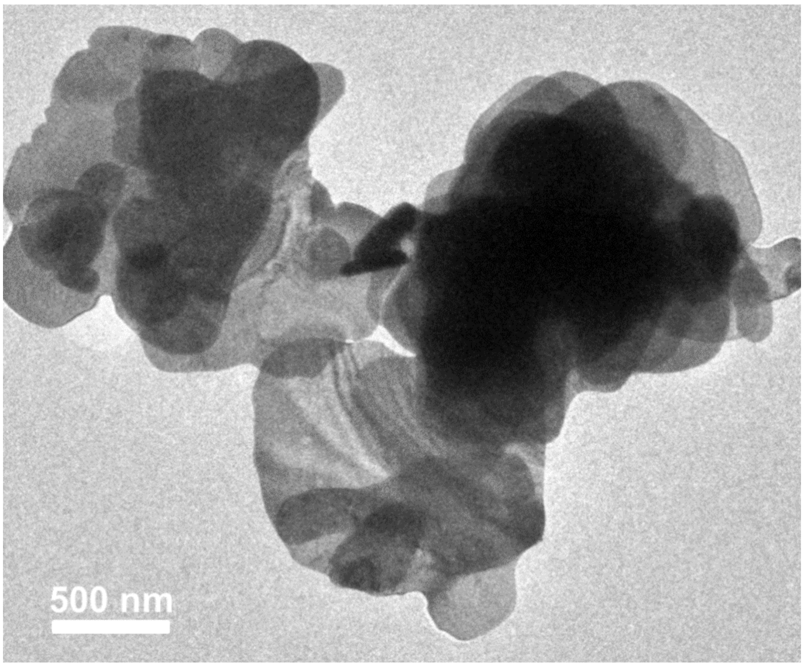

Supplementary Fig. 15 TEM image of BiOBr.

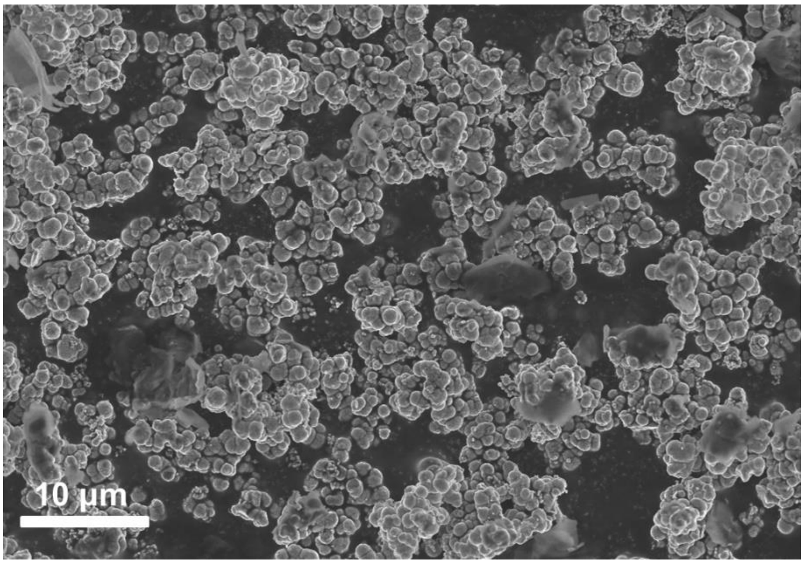

Supplementary Fig. 16 SEM image of $BaTiO_3$.

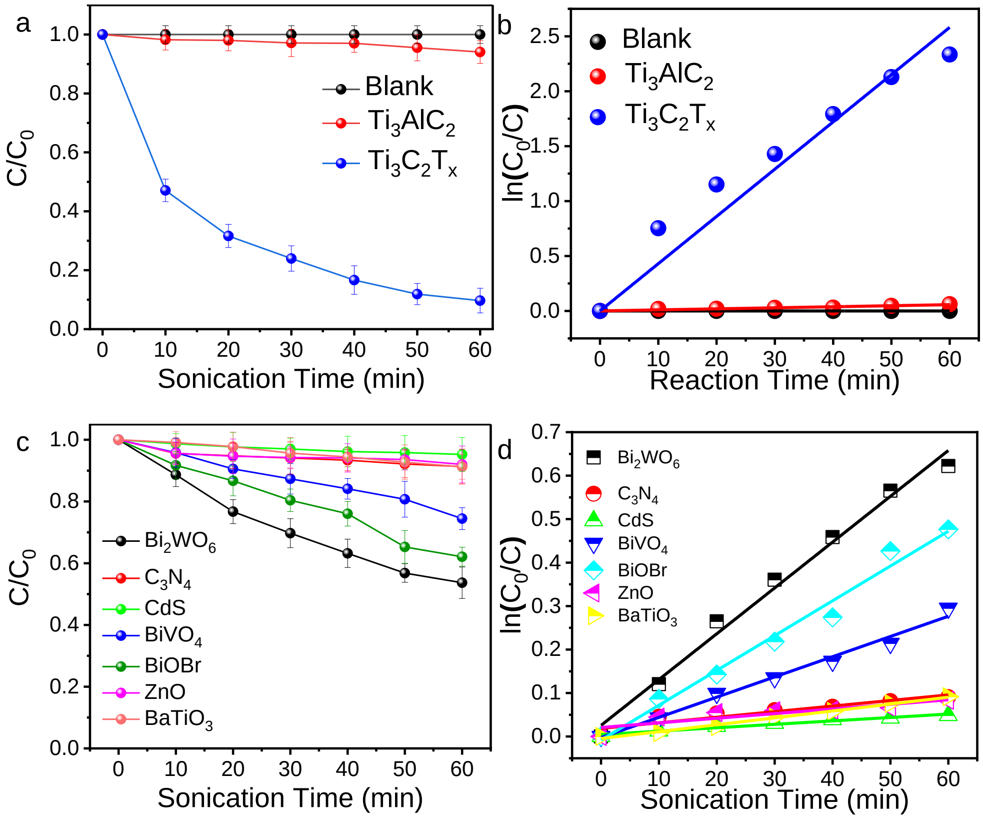

Supplementary Fig. 17 Piezocatalytic degradation of MB. a, b, c, d Piezocatalytic degradation of MB and the kinetic rate constant curves of different samples under sonication condition. All the data in (a–d) were collected for three times, and the error bars represent the standard deviation.

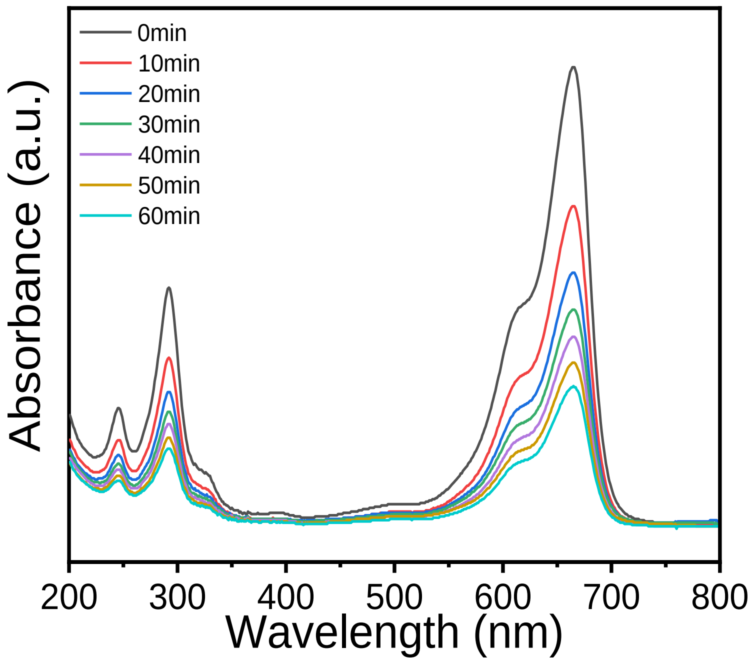

Supplementary Fig. 18 UV-vis absorption spectra of piezocatalytic degradation of MB solution with $Ti_3C_2T_X$ under continuous stirring (1000 rpm) at 298 K in the dark.

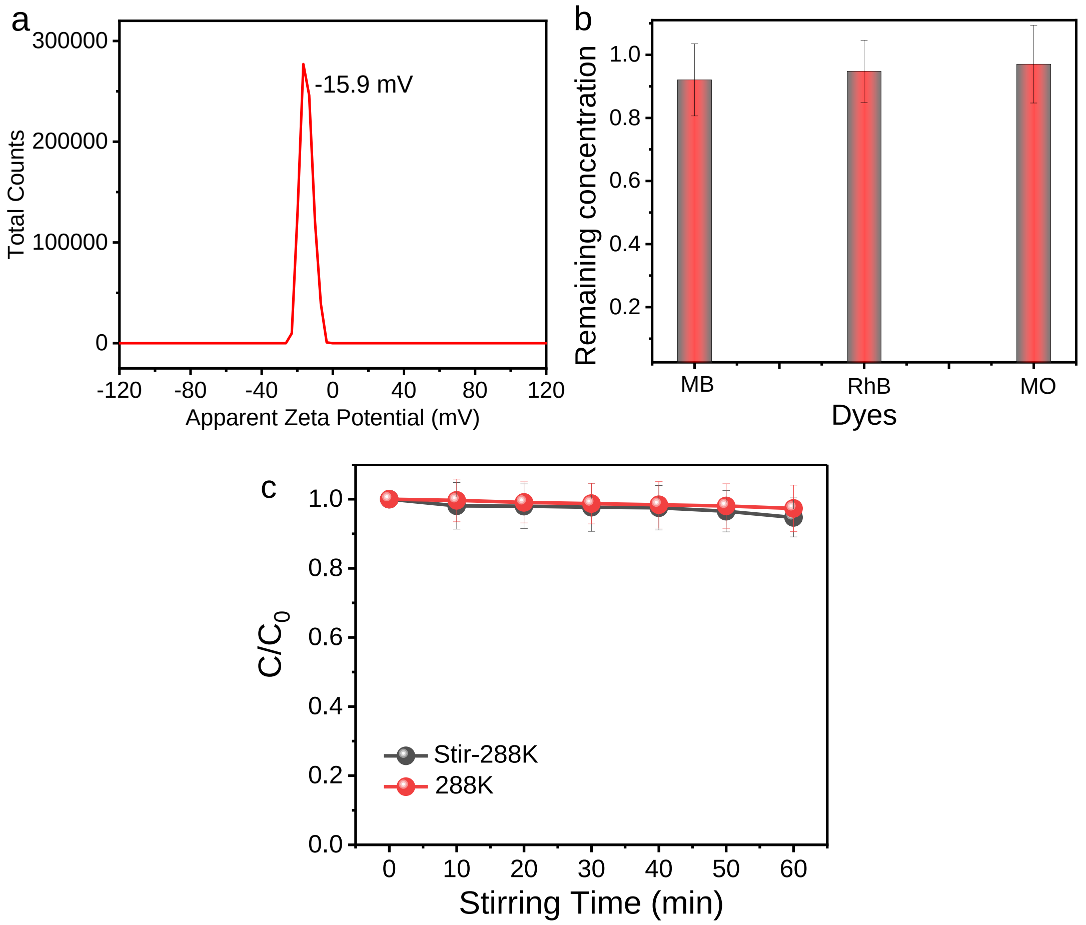

Supplementary Fig. 19 Adsorption property of the $Ti_3C_2T_X$. a Zeta potential of $Ti_3C_2T_X$. **b** Remaining concentration of dye solution after 1 hour pre-adsorption in the presence of $Ti_3C_2T_X$. **c** The adsorption curves for MB over the $Ti_3C_2T_X$ with or without stirring. All the data in (a–c) were collected for three times, and the error bars represent the standard deviation.

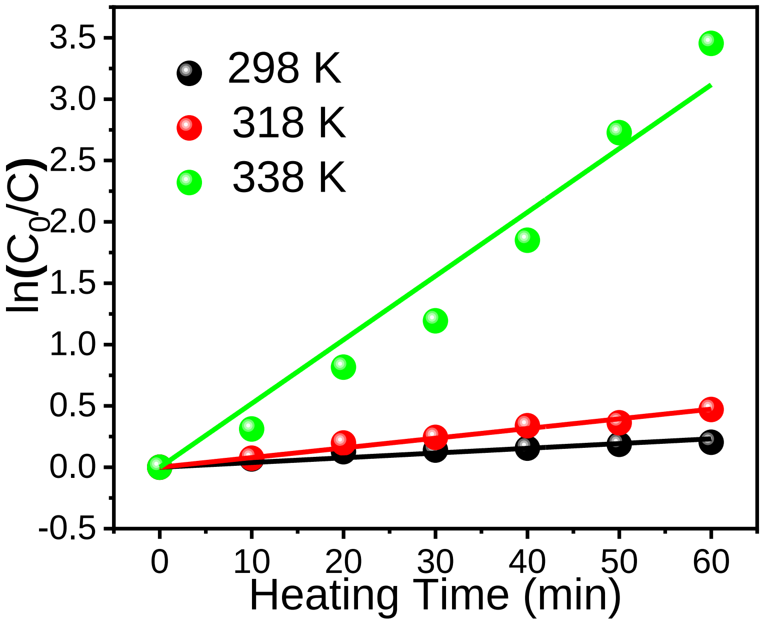

Supplementary Fig. 20 The kinetic rate constant curves for MB degradation over the $Ti_3C_2T_X$ at different temperature without stirring.

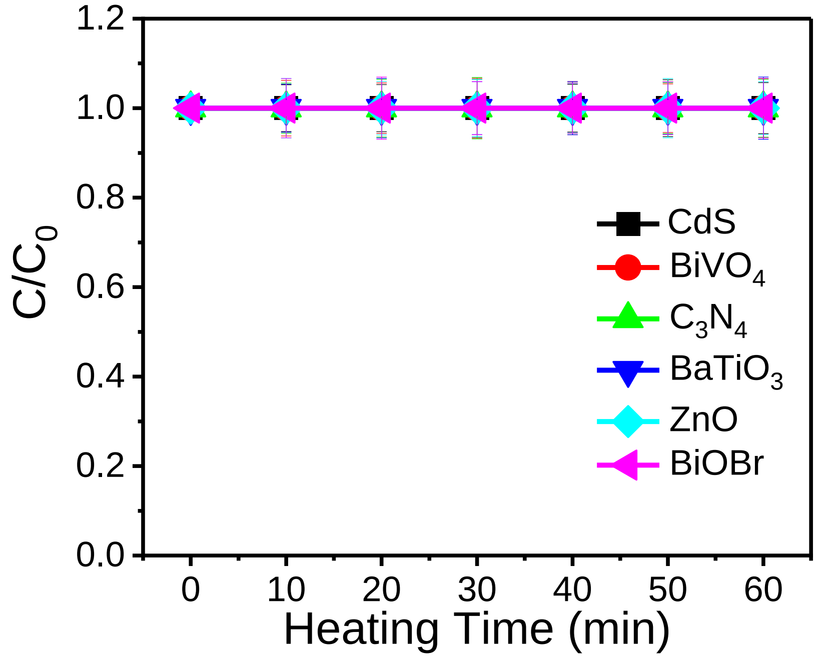

Supplementary Fig. 21 The catalytic degradation curves of MB using different semiconductors at 338 K in the dark. All the data in this figure were collected for three times, and the error bars represent the standard deviation.

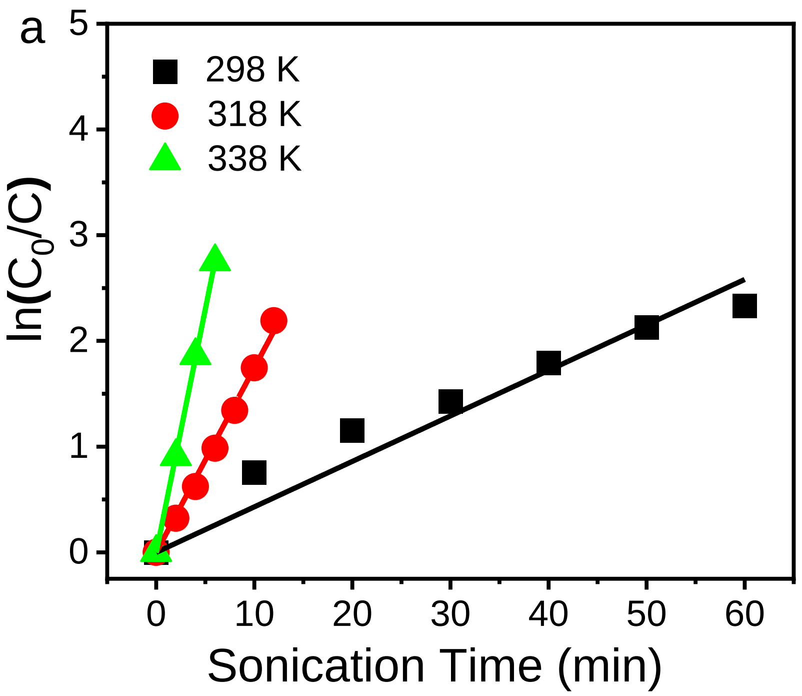

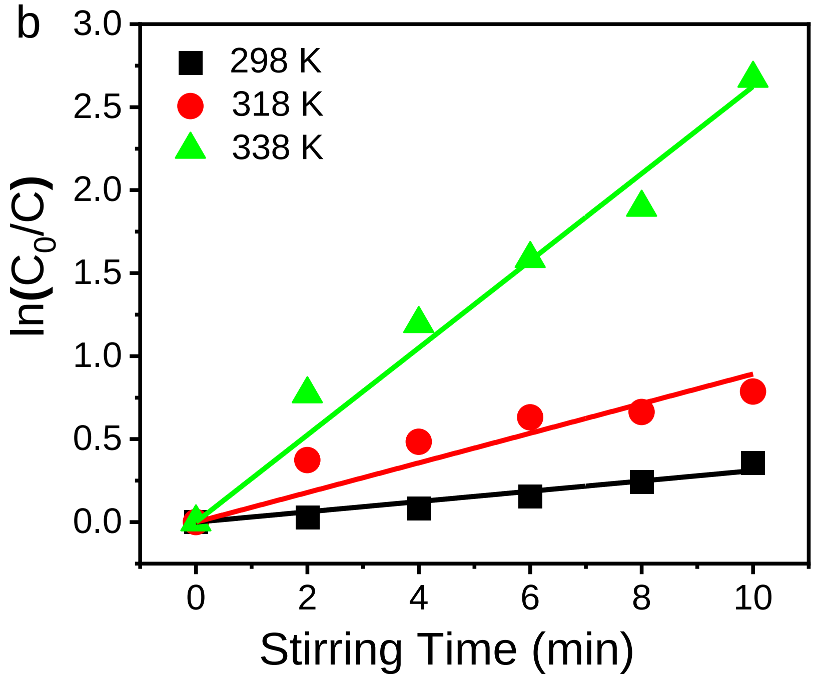

Supplementary Fig. 22 Kinetic curves for MB degradation. The kinetic rate constant curves for MB degradation over the $Ti_3C_2T_X$ at different temperatures under **a** sonication and **b** constant stirring (1000 rpm).

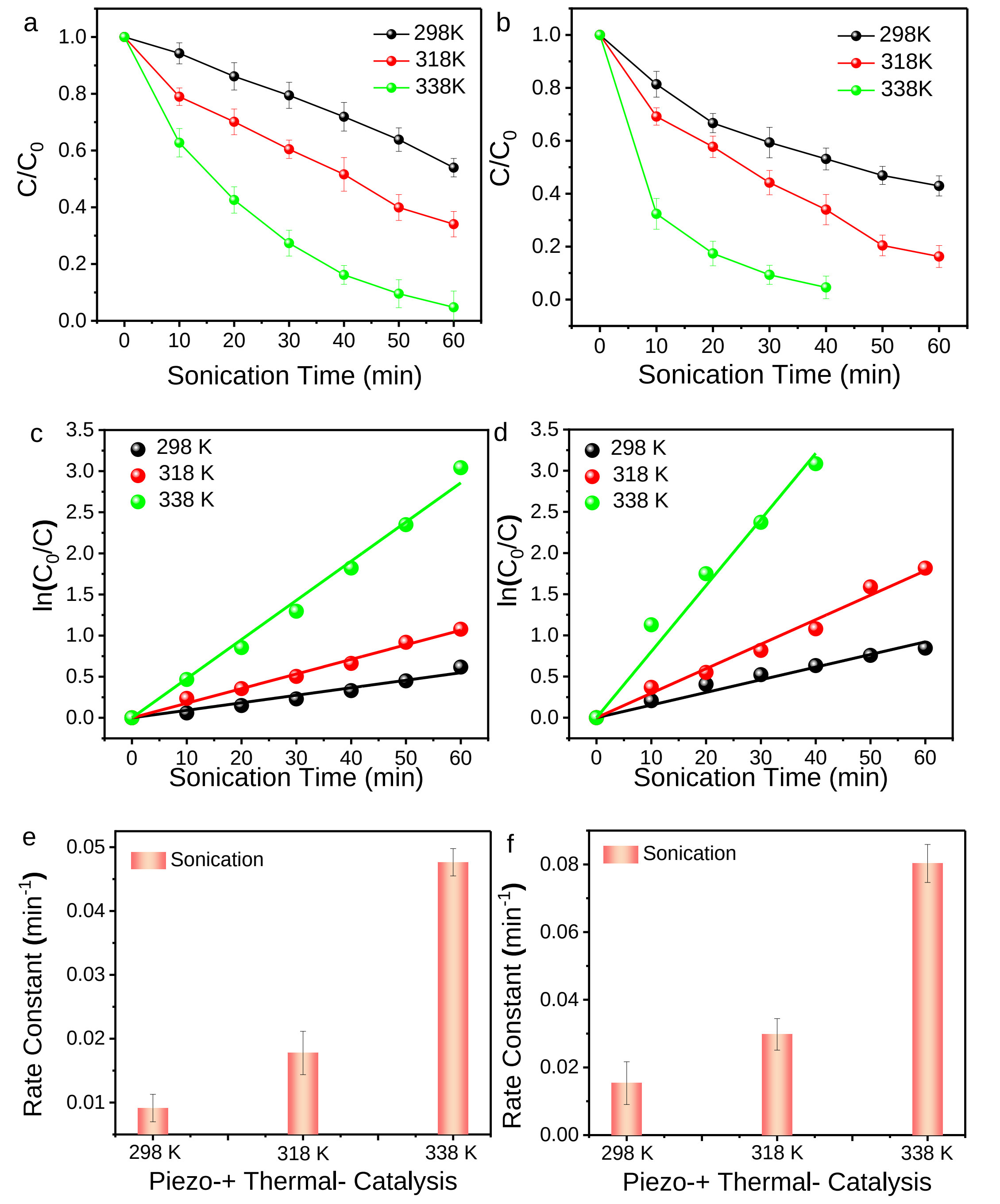

Supplementary Fig. 23 Catalytic degradation of dyes over the $Ti_3C_2T_X$. a Piezo-thermal catalytic degradation of methyl orange. **c** The kinetic rate constant curves and **e** the corresponding rate constants. **b** Piezo-thermal catalytic degradation of rhodamine B. **d** The kinetic rate constant curves and **f** the corresponding rate constants. All the data in (**a**, **b**, **e**, **f**) were collected for three times, and the error bars represent the standard deviation.

Supplementary Note 3: The piezo-thermal catalytic reactions for MO and RhB dyes degradation were investigated in **Supplementary Fig. 23**. It is clearly seen that the reaction rate increased with the increase of reaction temperature.

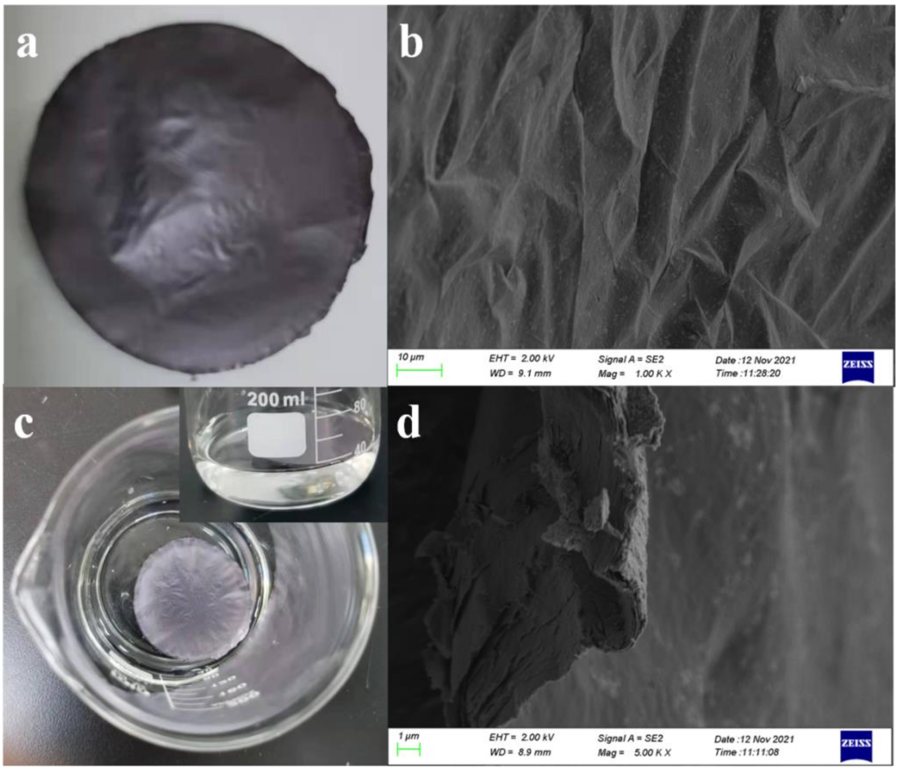

Supplementary Fig. 24 Characterization of $Ti_3C_2T_X$ film. a, c Photographs and **b, d** SEM images of $Ti_3C_2T_X$ film.

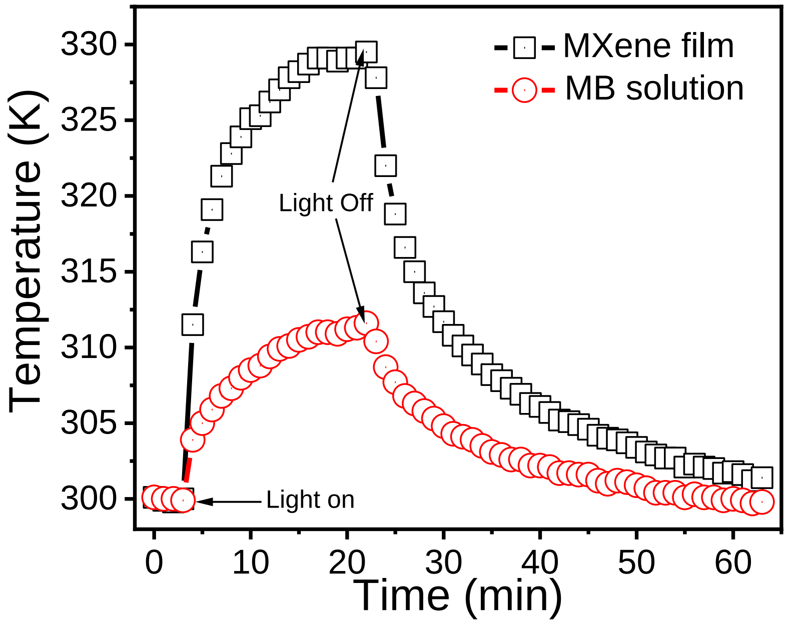

Supplementary Fig. 25 Photothermal test of the $Ti_3C_2T_X$ film floating on the MB solution.

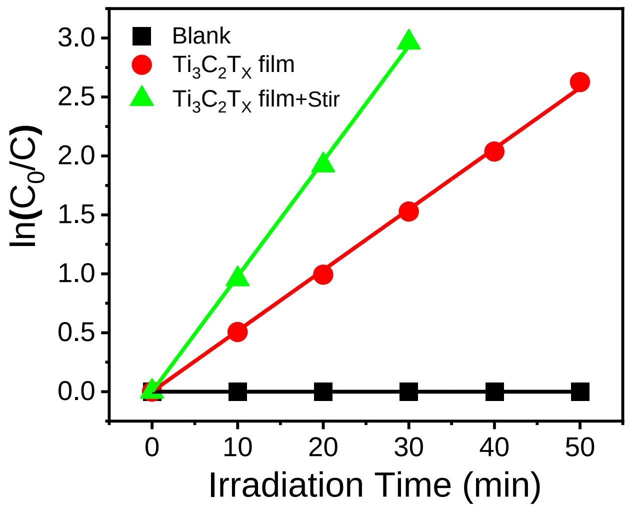

Supplementary Fig. 26 The kinetic rate constant curves for MB degradation over the $Ti_3C_2T_X$ film under NIR irradiation (700-1200 nm).

Supplementary Note 4: The rate constants for MB degradation over the $Ti_3C_2T_X$ film without and with stirring are determined to be 0.051 and 0.097 min^{-1}, respectively.

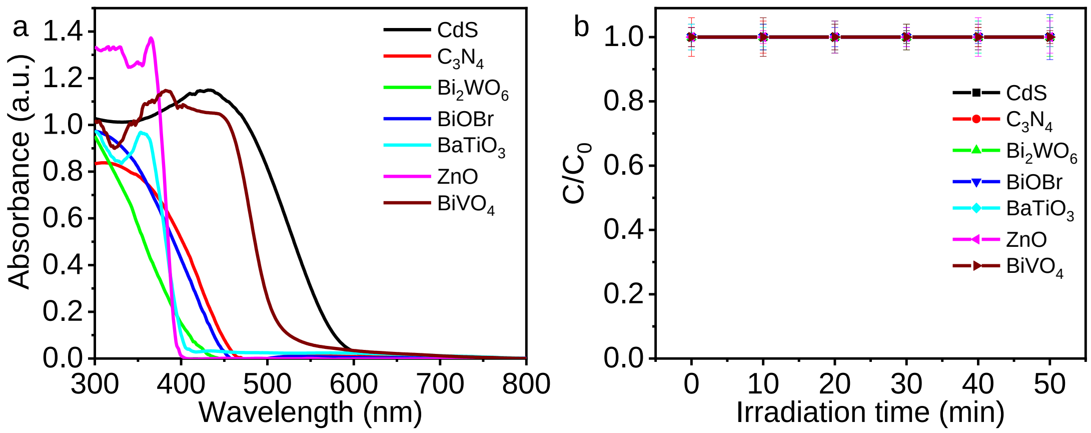

Supplementary Fig. 27 Optical and catalytic properties of the different semiconductor samples. a UV-vis diffuse reflectance spectra, and **b** NIR light-driven degradation of MB over the different polar semiconductors. All the data in (**b**) were collected for three times, and the error bars represent the standard deviation.

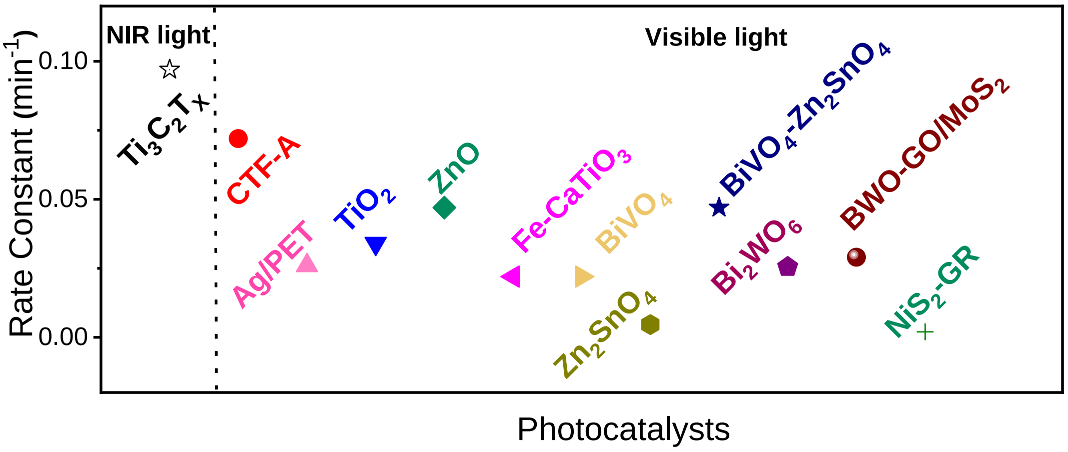

Supplementary Fig. 28 Light-driven degradation of MB over different samples.

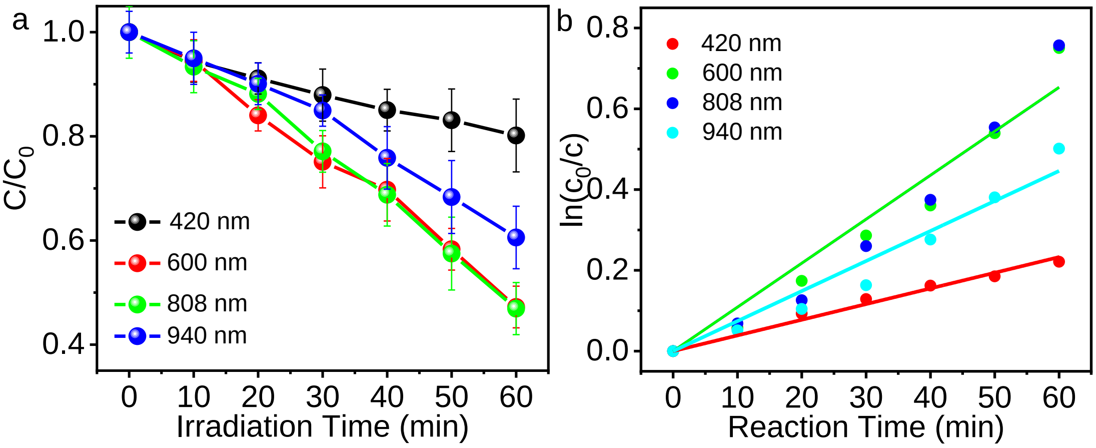

Supplementary Fig. 29 Photothermal catalytic degradation of MB over $Ti_3C_2T_X$ film. a, b Photothermal catalytic degradation of MB under different wavelengths of light irradiation over $Ti_3C_2T_X$ film. All the data in (a) were collected for three times, and the error bars represent the standard deviation.

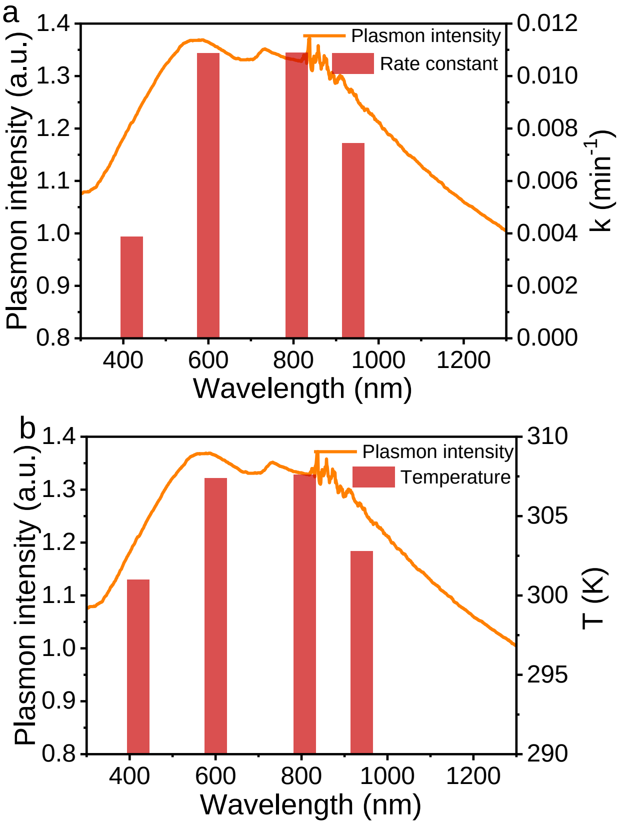

Supplementary Fig. 30 Photothermal catalytic properties of $Ti_3C_2T_X$ film. a Absorption spectrum and action spectrum of MB degradation of $Ti_3C_2T_X$ film. **b** Temperature of $Ti_3C_2T_X$ film under different wavelength of light irradiation.

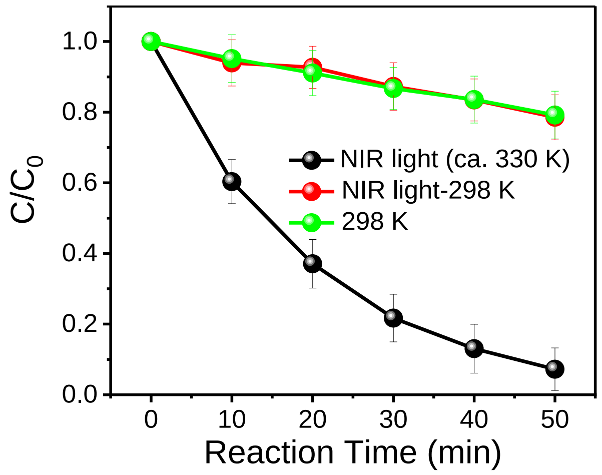

Supplementary Fig. 31 Catalytic degradation of MB under different conditions over $Ti_3C_2T_X$ film. All the data in this figure were collected for three times, and the error bars represent the standard deviation.

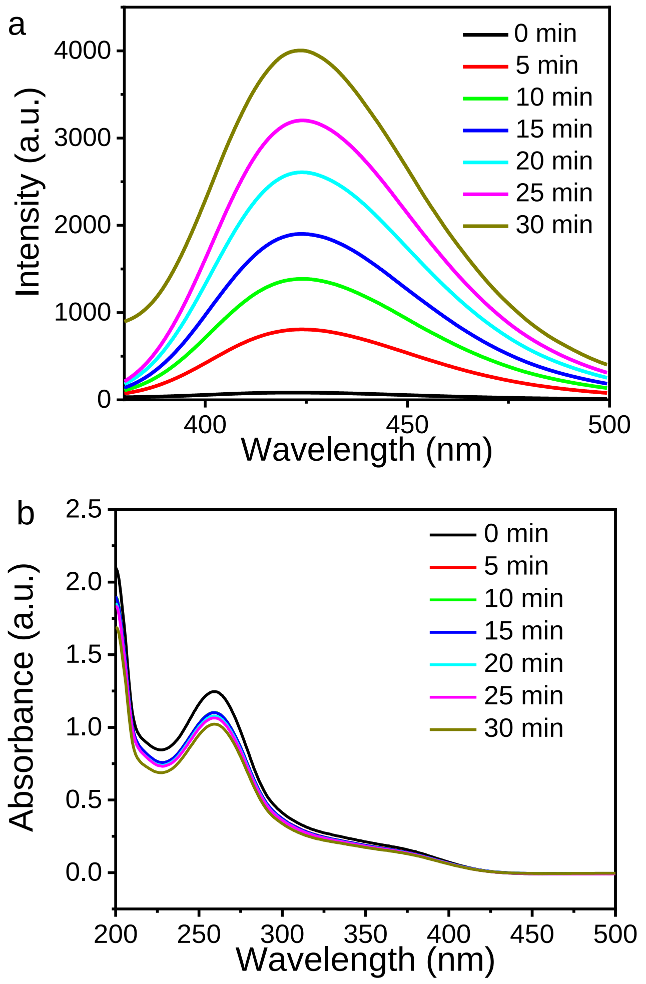

Supplementary Fig. 32 •OH and •O_2^- radicals generation under sonication. a Fluorescence spectra of TA solution over $Ti_3C_2T_X$ under sonication. **b** The absorbance of NBT solution over $Ti_3C_2T_X$ under sonication.

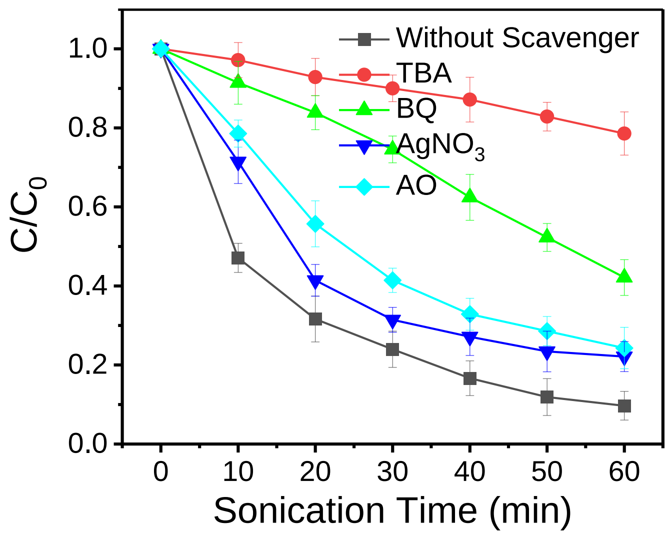

Supplementary Fig. 33 Piezocatalytic decomposition of MB with $Ti_3C_2T_X$ in the presence of different radical scavengers. All the data in this figure were collected for three times, and the error bars represent the standard deviation.

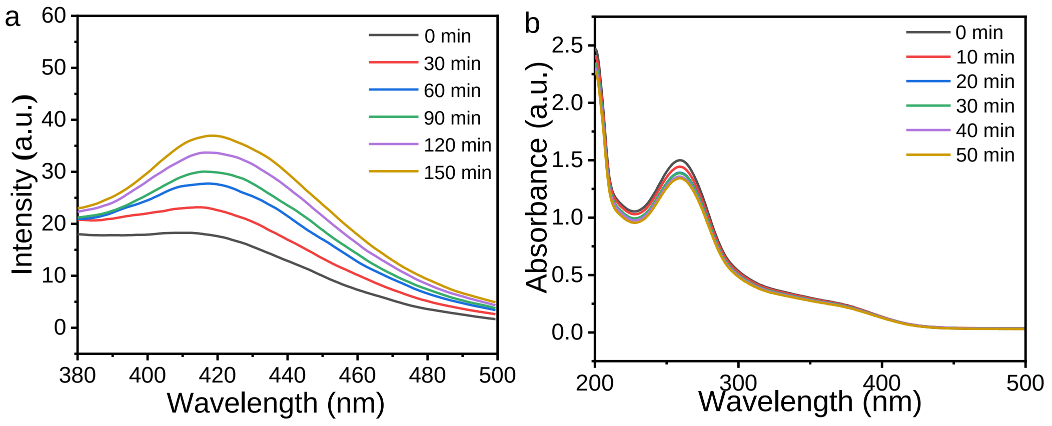

Supplementary Fig. 34 •OH and •O_2^- radicals generation at 338 K. a Fluorescence spectra of TA solution over $Ti_3C_2T_X$ without stirring in the dark at 338 K. **b** The absorbance of NBT solution over $Ti_3C_2T_X$ without stirring in the dark at 338 K.

Supplementary Note 5: Before reaction, TA is excited under 315 nm to produce an emission peak at ca. 350 nm. Therefore, the baseline at 0 minute is not flat (**Supplementary Fig. 34a**). After a period of reaction, TA reacts with •OH to produce hydroxyl terephthalic acid (HTA) with a PL peak at ca. 420 nm. When the peak intensity of the HTA is weak, the TA signal is noticeable. The interaction between the two PL peaks leads to the asymmetry of the HTA peak at 420 nm. However, when the intensity of the HTA is very strong (**Supplementary Fig. 32a**), the influence of the TA peak at 350 nm will be greatly weakened, thus resulting in the symmetry of the HTA peak.

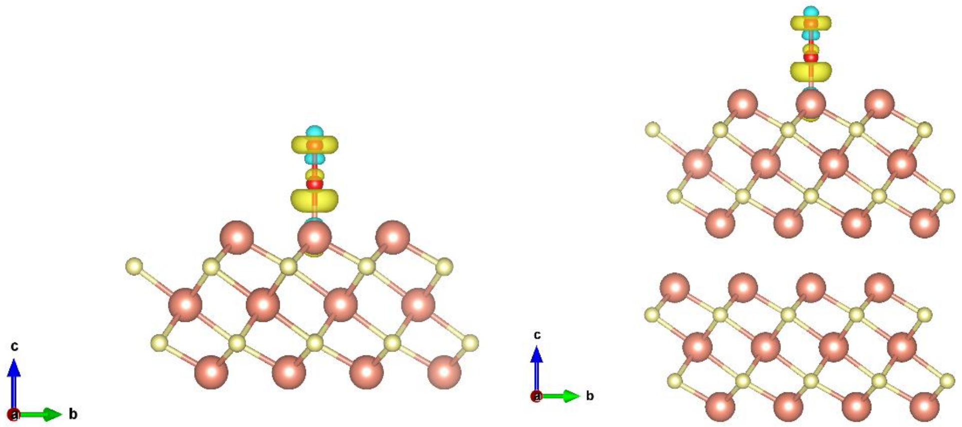

Supplementary Fig. 35 Configurations and charge density difference of O_2 adsorbed on the monolayer and bilayer $Ti_3C_2T_X$.

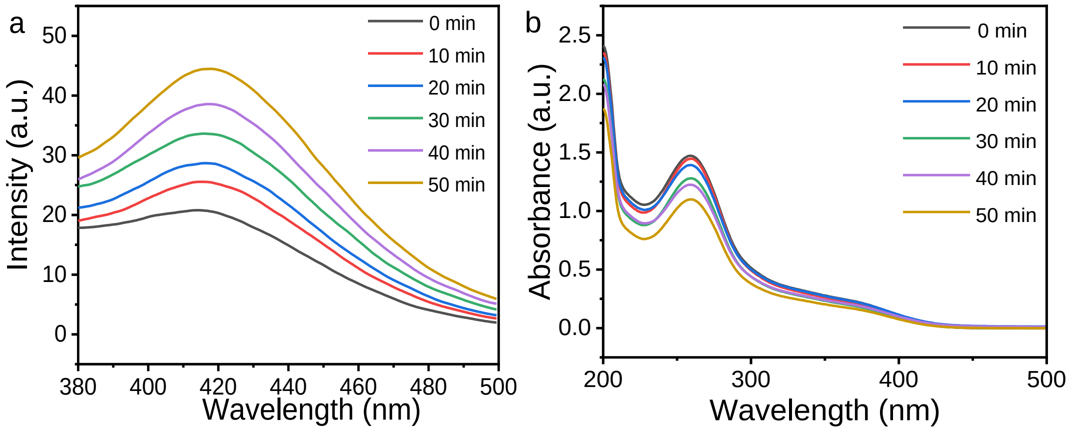

Supplementary Fig. 36 •OH and •O_2^- radicals generation under NIR light irradiation. a Fluorescence spectra of TA solution over $Ti_3C_2T_X$ under NIR light irradiation without stirring. **b** The absorbance of NBT solution over $Ti_3C_2T_X$ under NIR light irradiation without stirring.

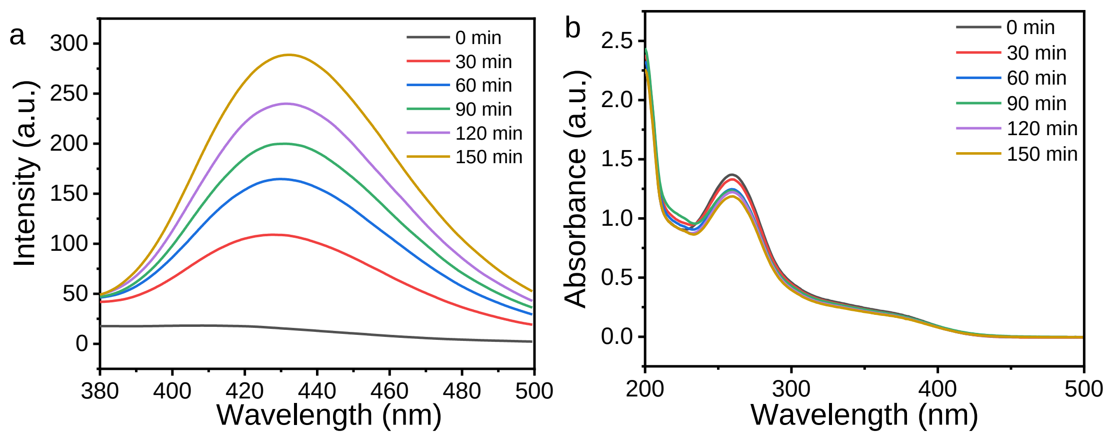

Supplementary Fig. 37 •OH and •O_2^- radicals generation under constant stirring. **a** Fluorescence spectra of TA solution over $Ti_3C_2T_X$ under continuous stirring in the dark at 298 K. **b** The absorbance of NBT solution over $Ti_3C_2T_X$ under continuous stirring in the dark at 298 K.

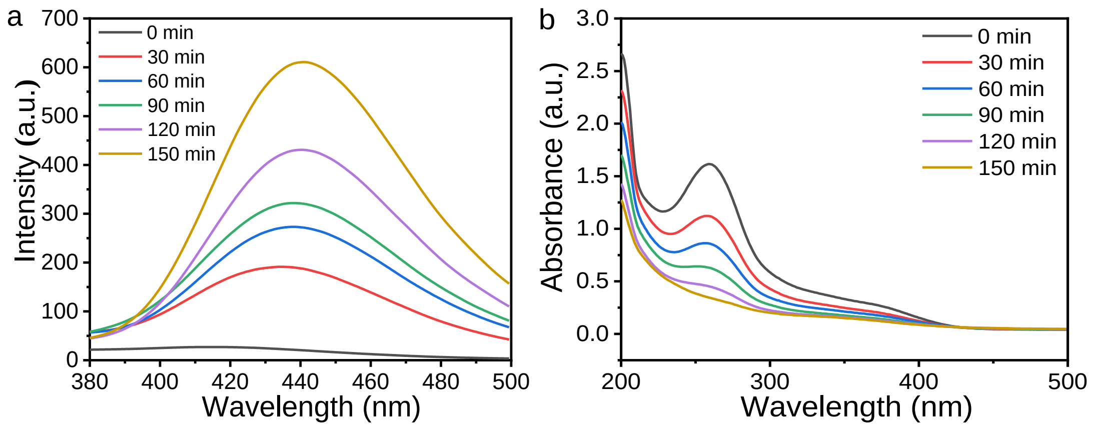

Supplementary Fig. 38 •OH and •O_2^- radicals generation under constant stirring at 338 K. **a** Fluorescence spectra of TA solution over $Ti_3C_2T_X$ under continuous stirring in the dark at 338 K. **b** The absorbance of NBT solution over $Ti_3C_2T_X$ under continuous stirring in the dark at 338 K.

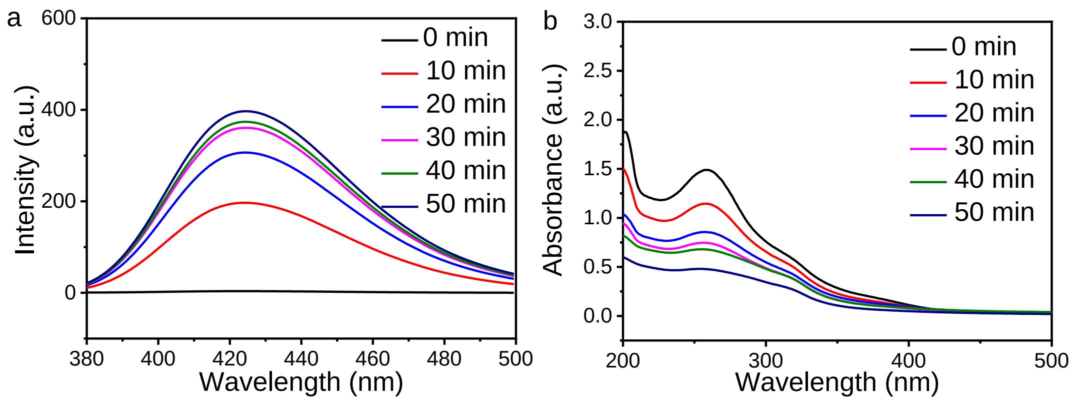

Supplementary Fig. 39 •OH and •O_2^- radicals generation under NIR light irradiation at 338 K. a Fluorescence spectra of TA solution over $Ti_3C_2T_X$ under NIR light irradiation with constant stirring (1000 rpm). **b** The absorbance of NBT solution over $Ti_3C_2T_X$ under NIR light irradiation with constant stirring (1000 rpm).

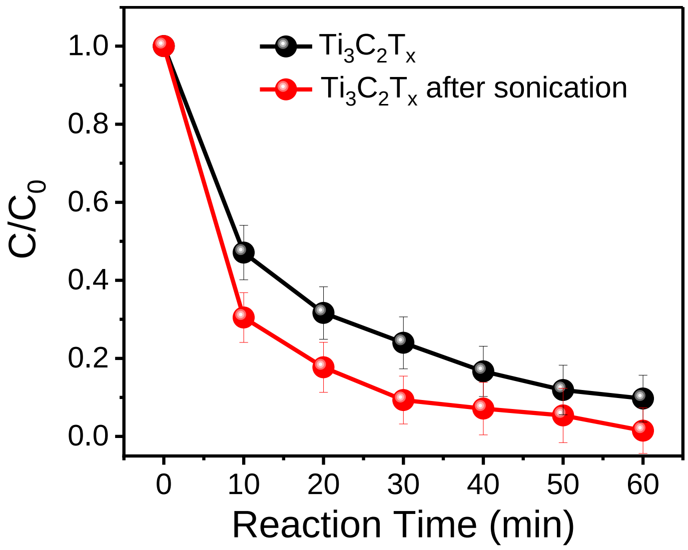

Supplementary Fig. 40 Piezocatalytic degradation of MB over the $Ti_3C_2T_X$ before and after sonication. All the data in this figure were collected for three times, and the error bars represent the standard deviation.

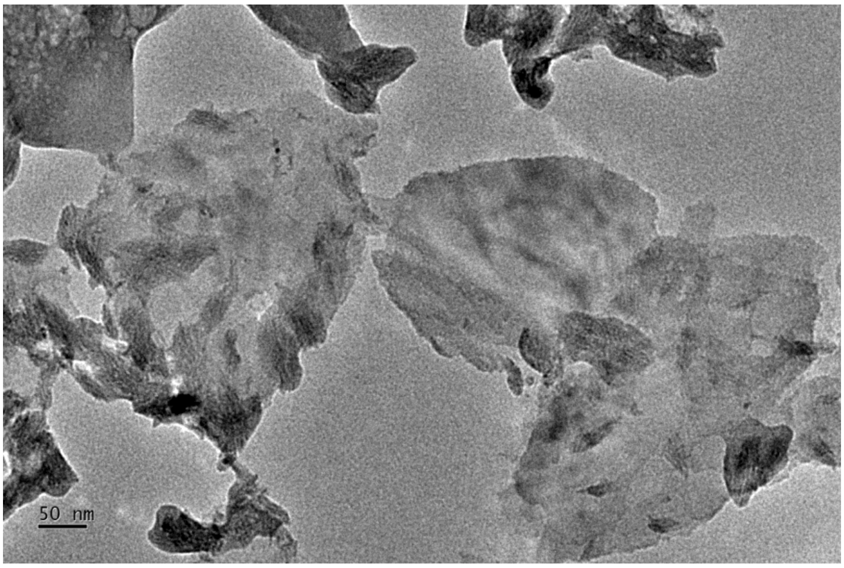

Supplementary Fig. 41 TEM image of $Ti_3C_2T_X$ after sonication.

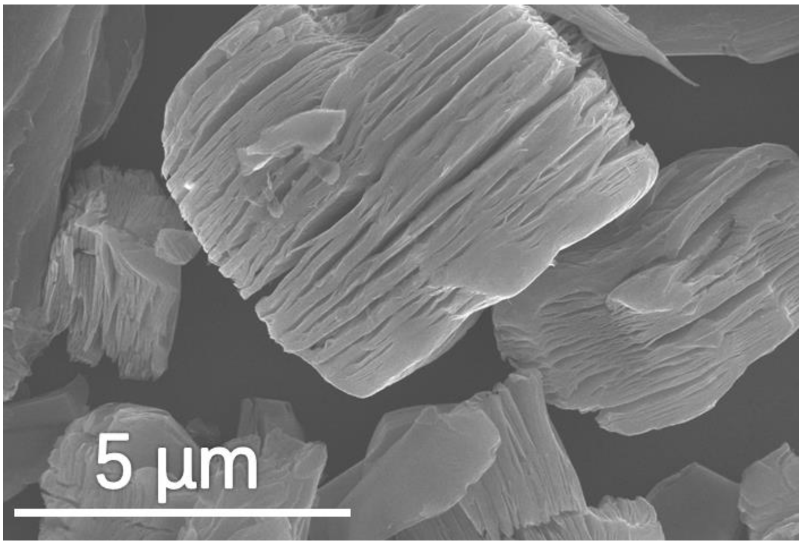

Supplementary Fig. 42 SEM image of $Ti_3C_2T_X$ before cycle reaction.

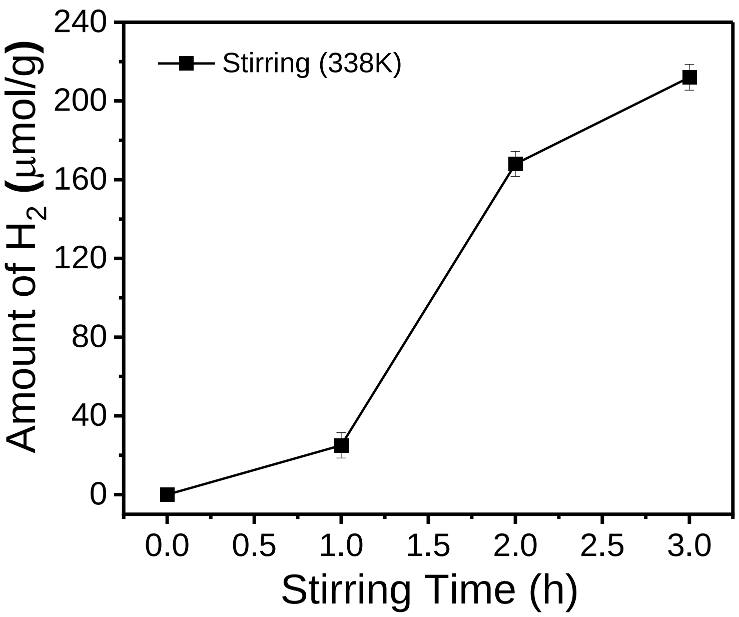

Supplementary Fig. 43 H_2 production of $Ti_3C_2T_X$ under stirring at 338 K. All the data in this figure were collected for three times, and the error bars represent the standard deviation.

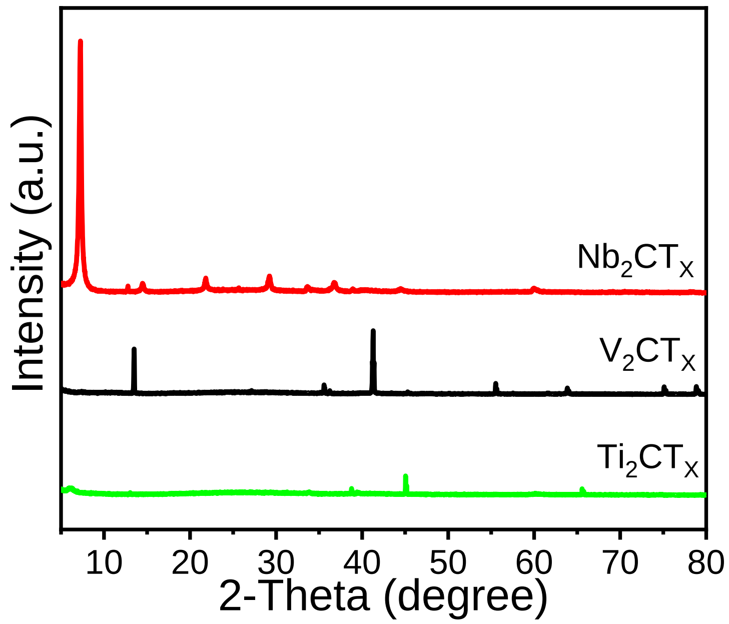

Supplementary Fig. 44 XRD patterns of the V_2CT_X, Nb_2CT_X and Ti_2CT_X.

Supplementary Note 7: These samples were prepared using the same procedure as $Ti_3C_2T_X$ except that the Ti_3AlC_2 was replaced by V_2AlC, Nb_2AlC or Ti_2AlC.

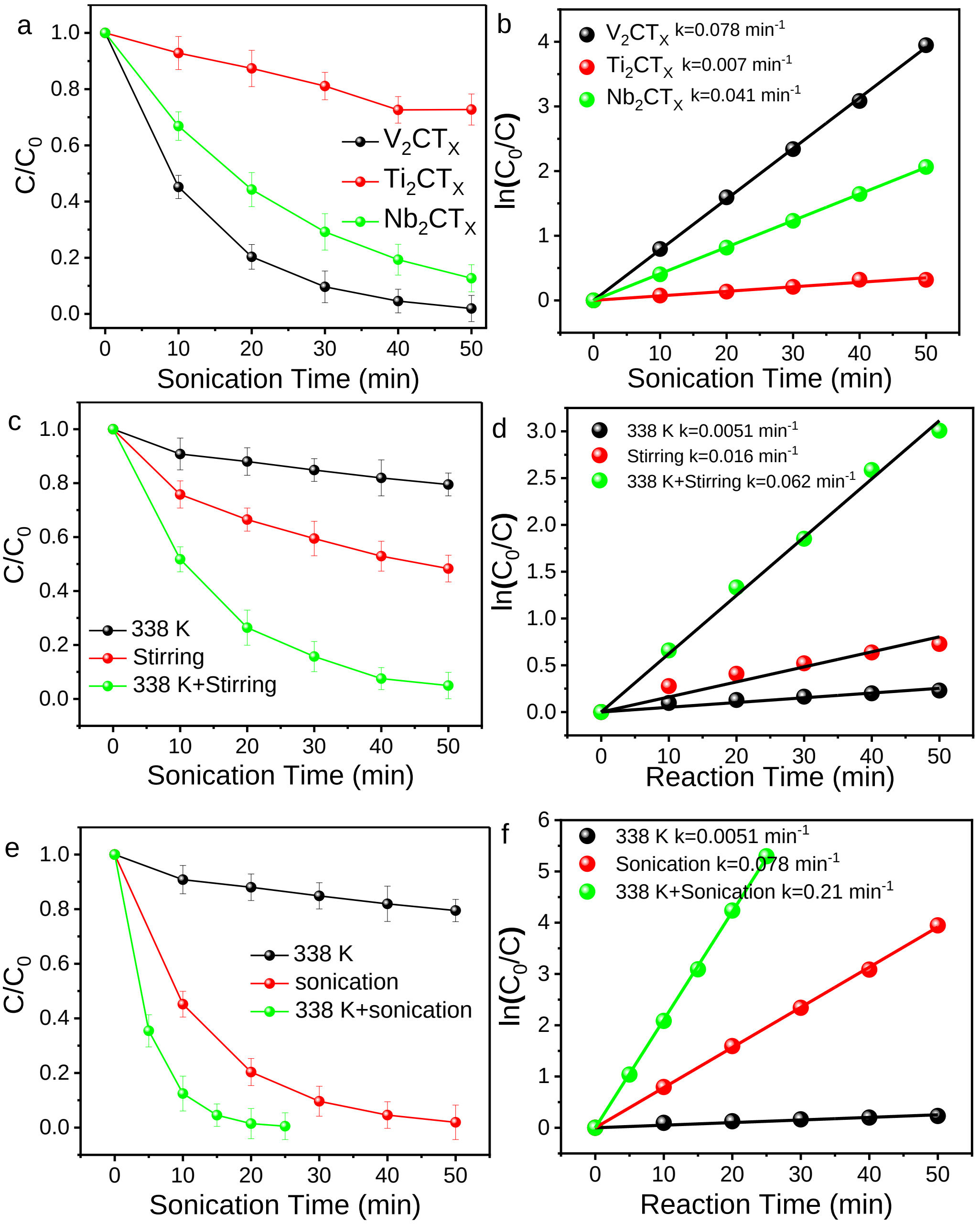

Supplementary Fig. 45 Catalytic degradation of MB over the Mxenes. a, c, e Catalytic degradation of MB over V_2CT_X, Nb_2CT_X and Ti_2CT_X MXenes under different conditions. **b, d, f** The corresponding reaction kinetics. All the data in (a, c, e) were collected for three times, and the error bars represent the standard deviation.

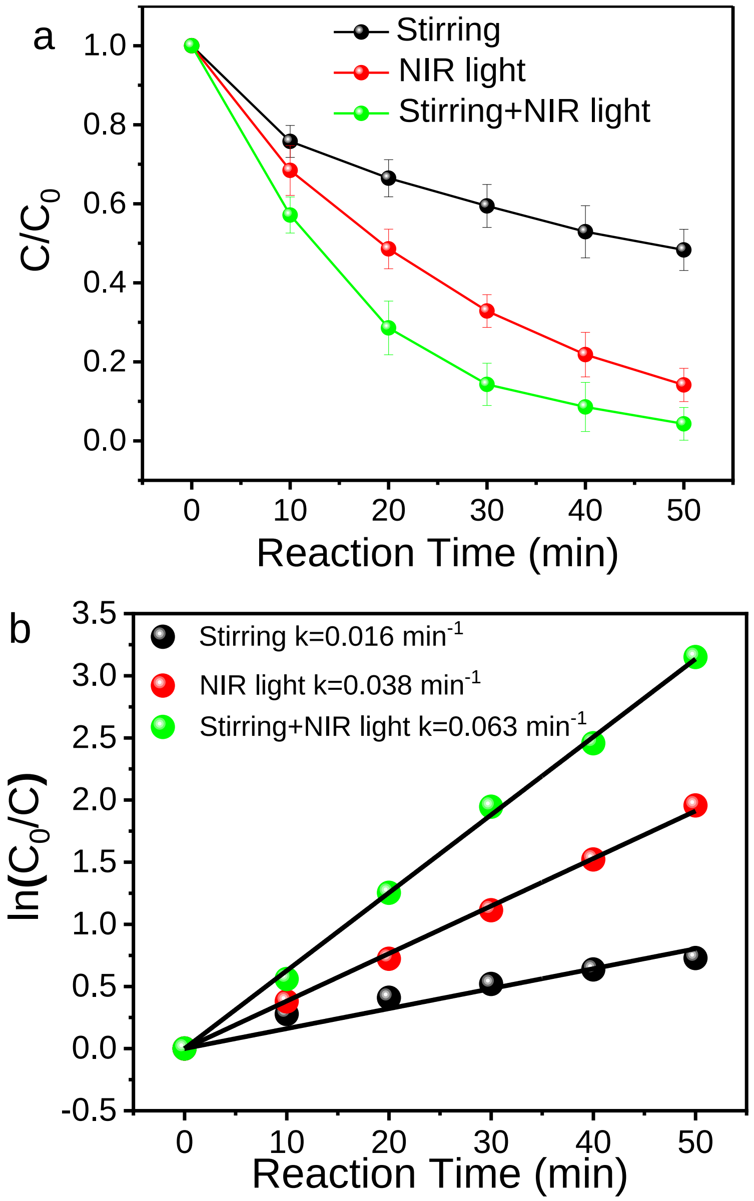

Supplementary Fig. 46 Catalytic degradation of MB over V_2CT_X. a Catalytic degradation of MB over V_2CT_X under different conditions. **b** The corresponding reaction kinetics. All the data in (a) were collected for three times, and the error bars represent the standard deviation.

Supplementary Table 1. Calculated dipole moments of Ti_3C_2 and $Ti_3C_2T_X$ monolayers.

Dipole moment	*x*(eÅ)	*y*(eÅ)	*z*(eÅ)
Ti_3C_2	0.000	0.000	0.000
$Ti_3C_2F_2$	-0.080	0.000	-0.088
$Ti_3C_2(OH)_2$	-0.154	0.000	0.038
Ti_3C_2OHF-1	-0.017	0.000	-0.253
Ti_3C_2OHF-2	-0.074	0.000	0.294
Ti_3C_2OHF-3	0.094	0.000	0.330
Ti_3C_2OHF-4	0.169	0.000	0.250

Supplementary Table 2. Calculated dipole moments of Ti_3C_2OHF-1 monolayer and Ti_3C_2OHF-1 multilayers (5 layers).

Dipole moment	*x*(eÅ)	*y*(eÅ)	*z*(eÅ)
Ti_3C_2OHF-1 monolayer	-0.017	0.000	-0.253
Ti_3C_2OHF-1 5 layers	-0.018	0.000	-0.238

Supplementary Note 1: the atomic structure of the Ti_3C_2OHF-1 is shown in the Supplementary Fig. 1.

Supplementary Table 3. The symmetry group of Ti_3C_2 and $Ti_3C_2T_X$ monolayers.

**Sample**	Space group	Symmetry
Ti_3C_2	$P\bar{3}m1$	centrosymmetric
$Ti_3C_2F_2$	P3m1	Non-centrosymmetric
$Ti_3C_2(OH)_2$	P3m1	Non-centrosymmetric
Ti_3C_2OHF-1	P3m1	Non-centrosymmetric
Ti_3C_2OHF-2	P3m1	Non-centrosymmetric
Ti_3C_2OHF-3	P3m1	Non-centrosymmetric
Ti_3C_2OHF-4	P3m1	Non-centrosymmetric

Supplementary Table 4. Comparison of piezocatalytic degradation of MB over different materials.

NO.	Sample	Energy Source	Sample Dosage	Rate Constant (min^{-1})	References
1	$Ti_3C_2T_X$	40 kHz 200 W	0.05 g Catalyst, 20 ppm, 50mL MB	0.037	This work
**2**	ZnO nanorods	40 kHz, 150 W	0.1 g Catalyst, 10 ppm, 100 mL MB	0.00341	5
**3**	ZnO	40 kHz, 300 W	0.2 g Catalyst, 10 ppm, 200 mL MB	0.01489	6
**4**	MoS_2/S, N doped graphene	40 kHz, 200 W	0.01 g Catalyst, 20 ppm, 20 mL MB	0.136	7
**5**	BCST particles	37 kHz, 200 W	0.01 g Catalyst, 20 ppm, 20 mL MB	0.01681	8
**6**	BCT-Sn	40 kHz, 200 W	0.2 g Catalyst, 5 ppm, 10 mL MB	0.00114	9
**7**	BCZT	40 kHz 70 W	0.1 g Catalyst, 8 ppm, 20 mL MB	0.00536	10
**8**	$BaTiO_3$ nanofibers	40 kHz 240 W	0.02 g Catalyst, 5 ppm, 20 mL MB	0.0537	11
**9**	Ag@$LiNbO_3$/PVDF	40 kHz 70 W	2.5 cm film 5 ppm, 10 mL MB	0.01777	12
**10**	$Sr_{0.8}Fe_{0.2}TiO_3$	40 kHz 70 W	2.5 cm film 3.6 ppm, 10 mL MB	0.0044	13

Supplementary Table 5. The BET surface areas of the samples.

Sample	S_{BET} (m^2/g)
Ti_3AlC_2	1.6
$Ti_3C_2T_x$	2.8
ZnO	25.1
$BiVO_4$	1.4
C_3N_4	39.5
$BaTiO_3$	4.5
BiOBr	2.4
Bi_2WO_6	30.3
CdS	7.5

Supplementary Table 6. Comparison of light-driven MB degradation by photocatalysis over some typical semiconductors and photothermal catalysis over $Ti_3C_2T_X$ conductor.

NO.	Sample	Light Source	Reaction conditions	Rate Constant (min^{-1})	References
1	$Ti_3C_2T_X$	NIR light (700-1200 nm)	0.015 g catalyst, 30 mL of 20 ppm MB	0.051 (without stirring), 0.097 (with stirring) 30 min degradation ratio 99% (with stirring)	This work
**2**	$BiPO_4:Yb^{3+},Tm^{3+}/BiVO_4$	> 400 nm, Xe lamp	0.03 g catalyst, 30 mL of 5 × 10^{-5} mol L^{-1} MB	0.01	14
**3**	$BiPO_4:Yb^{3+},Tm^{3+}/BiVO_4$	800 > λ > 300 nm, Xe lamp	0.03 g catalyst, 30 mL of 5 × 10^{-5} mol L^{-1} MB	0.047	14
**4**	$BiPO_4:Yb^{3+},Tm^{3+}/BiVO_4$	980 nm, NIR laser	0.03 g catalyst, 30 mL of 5 × 10^{-5} mol L^{-1} MB	12 h degradation ratio 40%	14
**5**	$NaYF_4$:Yb,Tm @TiO_2/RGO	980 nm, diode laser	0.005 g catalyst, 5 mL of 10 ppm MB	0.0023 min^{-1}	15
**6**	$NaYF_4$:Yb,Tm @TiO_2	980 nm laser	0.0005 g catalyst, 0.5 mL of 15 ppm MB	14 h degradation ratio 65%	16
**7**	β-$NaYF_4:Yb^{3+}$, Tm^{3+}@TiO_2	980 nm laser	0.005 g catalyst, 0.5 mL of 15 ppm MB	24 h degradation ratio 68%	17
**8**	$NaYF_4$:Yb,Tm-BiOCl	300 W Xe lamp, λ > 780 nm,	0.02 g catalyst, 50 mL of 2 × 10^{-5}mol L^{-1} MB	160 min degradation ratio 60%	18
**9**	CdS-C-$NaYF_4$:Yb,Tm	Xe lamp Vis bandpass (400-780 nm) and NIR band pass (780-2500 nm)	0.01 g catalyst, 50 mL of 5 × 10^{-5}mol L^{-1} MB	60 min degradation ratio 85%; 52%	19
**10**	$NaYF_4$ UCMPs	Visible light, 980 nm laser fiber	0.0025 g catalyst, 5 mL of 5 ppm	120 min degradation	20

			MB	ratio 52%; 5 h degradation ratio 45%	
**11**	Er^{3+}/Yb^{3+} codoped (CaF_2@TiO_2)	Xe lamp (720 nm < λ < 1100 nm)	0.05 g catalyst, 50 mL of 10 ppm MB	12 h degradation ratio 60%	21
**12**	$NaYF_4$: Yb,Er/CdSe	1560 nm and 980 nm laser	0.0005 g catalyst, 0.5 mL of 15 ppm MB	3.5×10^{-4} (min^{-1}) and 8.0×10^{-4} (min^{-1})	22
**13**	NYT-15/C_3N_4	980 nm laser	0.001 g catalyst, 0.5 mL of 15 ppm MB	2 h degradation ratio 53%	2
**14**	Bi_2S_3/C-dots	Xe lamp, vis- (420 nm < λ < 780 nm) and NIR- (λ > 800 nm)	0.05 g catalyst, 50 mL of 10 ppm MB	60 min degradation ratio 99%, 87%	23
**15**	BiOBr:$Yb^{3+}/Er^{3+}/Ho^{3+}$	Xe lamp, simulated sunlight (300 nm < λ< 1100 nm)	0.01 g catalyst, 30 mL of 10 ppm MB	120 min degradation ratio 78%, 87%	24

Supplementary Table 7. Bader charge transfer and charge density difference of O_2 adsorbed on $Ti_3C_2T_X$.

**Atom**	**Molecule**	**Monolayer**	**Bilayer**
**O1**	5.97	6.54	6.28
**O2**	6.01	6.28	6.53
**Total bader charge difference**	—	0.85	0.83
**O-O (Å)**	1.23	1.31	1.31
**O-Ti (Å)**	—	1.83	1.82
**E-ad (eV)**	—	-3.622	-3.557

Supplementary Table 8. Comparison of piezocatalytic H_2 production over different materials.

NO.	Sample	Energy Source	Reaction Conditions	H_2 production Rate (μmol/g/h)	References
1	$Ti_3C_2T_X$	40 kHz, 200 W	5 mg catalyst, 10 mL of CH_3OH solution (10v%)	1341	This work
**2**	MoS_2 nanosheets<br>WS_2 nanosheets<br>WSe_2 nanosheets	40 kHz, 110 W	20 mg catalyst, 100 mL of $FeSO_4$ solution (0.01 M)	29<br>15<br>11	25
**3**	Bi_2WO_6 nanoplates	40 kHz, -	20 mg catalyst, 10 mL of TEOA solution (20%v/v)	191	26
**4**	$CH_3NH_3PbI_3$	70 W, -	50 mg catalyst, 5.5 mL of HI solution (57 wt%)	4	27
**5**	$0.7BiFeO_3/0.3 BaTiO_3$	40 kHz, 100 W	30 mg catalyst, 50 mL of H_2O CH_3OH solution (10%)	1322	28
**6**	$BaTiO_3$	40 kHz, 100 W	10 mg catalyst, 100 mL of TEOA (15v%)	92	29
**7**	$BiFeO_3$	40 kHz, 100 W	10 mg catalyst, 10 mL of Na_2SO_3 solution (0.05 M)	124	30
**8**	Pd-$BiFeO_3$	40 kHz, 100 W	10 mg catalyst, 10 mL of Na_2SO_3 solution (0.05 M)	1140	31
**9**	TiO_2/ZnO nanowires	ultrasonic: 50 W, light:50 W	220 mg catalyst, 150mL of CH_3OH solution (20v%)	3	32
**10**	CdS	100 W, 45 kHz, light: 0.88 mW/cm^2	50 mg catalyst, 80 mL of lactic acid solution (10v%)	284	33
**11**	Ni/GaN nanowires	110 W, 40 kHz	2 mg catalyst, 100 mL of TEOA solution (15v%)	88	34
**12**	$Sr_{0.5}Ba_{0.5}Nb_2O_6$-500 ℃	110 W, 40 kHz	10 mg catalyst,	109	35

		100 mL of TEOA solution (15v%)				

Supplementary Table 9. The material cost ($C_{material}$) and energy cost (C_{energy}) for the preparation of different piezocatalysts.

Sample	Materials (CNY ¥)			Yield	Synthesis	Total cost	Ref.
**$Ti_3C_2T_X$**	Ti_3AlC_2	1.00 g	1.80 ¥	0.86 g	308 K 24 h (Heat Stirrer)	5.58 ¥/g	This work
	LiF	1.00 g	1.90 ¥				
	HCl	20.00 mL	0.60 ¥		C_{energy}: 0.50 ¥		
	$C_{material}$: 4.30 ¥						
**MoS_2**	NaCl	3.75 g	0.08 ¥	0.16 g	493 K 24 h (Oven)	137.44 ¥/g	27
	NaOH	24.00 g	1.15 ¥				
	SiO_2	2.4.00 g	0.23 ¥				
	MoO_3	0.144 g	0.15 ¥				
	$NH_3 \cdot H_2O$	30.00 ml	0.84 ¥		C_{energy}: 18.48 ¥		
	HCl	30.00 ml	1.02 ¥				
	CH_4N_2S	0.40 g	0.04 ¥				
	$C_{material}$: 3.51 ¥						
**Bi_2WO_6**	$Bi(NO_3)_3 \cdot 5H_2O$	1.00 mmol	0.33 ¥	0.349 g	433K 20h (Oven)	46.48 ¥/g	28
	Na_2WO_4	1.00 mmol	0.49 ¥		C_{energy}: 15.4 ¥		
	$C_{material}$: 0.82 ¥						
**$MAPbI_3$**	MAI	1.00 mmol	10.43 ¥	0.62 g	343 K 12h (Oven)	52.03 ¥/g	29
	PbI_2	1.00 mmol	1.38 ¥				
	C_3H_7NO	100.00 ml	9.00 ¥				
	C_6H_5Cl	20.00 ml	2.21 ¥				
	$C_{material}$: 23.02 ¥				C_{energy}: 9.24 ¥		
**$0.7BiFeO_3$/ $0.3BaTiO_3$**	$Bi(NO_3)_3 \cdot 5H_2O$	0.51 g	0.19 ¥	4.28 g	473 K 8h (Oven)	1.55 ¥/g	30
	$Fe(NO_3)_3 \cdot 5H_2O$	0.42 g	0.03 ¥				
	HNO_3	2.00 ml	0.10 ¥				
	KOH	15.00 ml	0.10 ¥		C_{energy}: 6.16 ¥		
	$Ti(OC_4H_9)_4$	0.15 ml	0.04 ¥				
	$C_{material}$: 0.46 ¥						
**$BaTiO_3$**	NaOH	10.00 mol	19.20 ¥	5.22 g	483 K 24h (Oven)	9.25 ¥/g	31
	TiO_2	1.88 g	9.14 ¥				
	HCl	0.20 mol	0.54 ¥				
	$Ba(OH)_2 \cdot 8H_2O$	0.04 mol	0.91 ¥		C_{energy}: 18.48 ¥		
	$C_{material}$: 29.79 ¥						

**$BiFeO_3$**	$Bi(NO_3)_3 \cdot 5H_2O$	2.43 g	0.92 ¥	1.57 g	453 K 48h (Oven) 773 K 2h (Muffle furnace)	30.99 ¥/g	32
	$(CH_2OH)_2$	100.00 ml	3.70 ¥				
	$FeCl_3 \cdot 5H_2O$	1.35 g	0.26 ¥				
	$NH_3 \cdot H_2O$	50.00 ml	1.40 ¥		C_{energy}: 40.46 ¥		
	NaOH	0.2 mol	1.92 ¥				
	$C_{material}$: 8.20 ¥						
**Pd-$BiFeO_3$**	$BiFeO_3$	0.01 g	0.30 ¥	0.0166 g	423 K 3h (Oven)	180.12 ¥/g	33
	Pd-NCS	2.4 mmol	0.38 ¥		C_{energy}: 2.31 ¥		
	$C_{material}$: 0.68 ¥						

Supplementary Note 6: The energy cost for material synthesis is calculated based on the rated power for the instrument (water bath heated magnetic stirrer: 30 W; oven: 1100 W; muffle furnace: 2500 W).

Supplementary Table 10. The energy cost for catalytic processes.

Reaction condition	Instrument	Power (W)	Cost (1 hour)
**Sonication**	Ultrasonic cleaner	200	0.14 ¥
**Stir**	Stirrer	12	0.0084 ¥
**Heating**	Water bath heated magnetic stirrer	30	0.021 ¥
**NIR light**	NIR lamp	100	0.070 ¥

Supplementary References.

1. Hafner J. Ab-initio simulations of materials using VASP: Density-functional theory and beyond. *J. Comput. Chem.* **29**, 2044-2078 (2008).
2. Perdew, J. P.; Wang, Y., Accurate and simple analytic representation of the electron-gas correlation energy. *Phys. Rev. B* **23,** 13244-13249 (1992).
3. Grimme S, Antony J, Ehrlich S, Krieg H. A consistent and accurate ab initio parametrization of density functional dispersion correction (DFT-D) for the 94 elements H-Pu. *J. Chem. Phys.* **132**, 154104 (2010).
4. Pan Y, Xu H, Chen M, Wu K, Zhang Y, Long D. Unveiling the Nature of Room-Temperature O_2 Activation and $O_2^{\bullet-}$ Enrichment on MgO-Loaded Porous Carbons with Efficient H_2S Oxidation. *ACS Catal.* **11**, 5974-5983 (2021).
5. Ning X, Hao A, Cao Y, Hu J, Xie J, Jia D. Effective promoting piezocatalytic property of zinc oxide for degradation of organic pollutants and insight into piezocatalytic mechanism. *J. Colloid Interface Sci.* **577**, 290-299 (2020).
6. Peng F, *et al.* A discovery of field-controlling selective adsorption for micro ZnO rods with unexpected piezoelectric catalytic performance. *Appl. Surf. Sci.* **545**, 149032 (2021).
7. Pan M, Liu S, Chew JW. Unlocking the high redox activity of MoS_2 on dual-doped graphene as a superior piezocatalyst. *Nano Energy* **68**, 104366 (2020).
8. Maria Joseph Raj NP, Alluri N, Khandelwal G, Kim S-J. The Morphotropic Phase Boundary based BCST Ferroelectric System for Water Remediation through Bi-catalytic Activity. *J. Alloys. Compd.* **871**, 159503 (2021).
9. Qifeng L, Jingjun M, Sharma M, Vaish R. Photocatalytic, piezocatalytic and piezo-photocatalytic effects in ferroelectric $(Ba_{0.875}Ca_{0.125})(Ti_{0.95}Sn_{0.05})O_3$ ceramics. *J. Am. Ceram. Soc.* **102**, 159503 (2019).
10. Sharma M, Vaish R. Vibration energy harvesting for degradation of dye and bacterial cells using cement-based $Ba_{0.85}Ca_{0.15}Zr_{0.1}Ti_{0.90}O_3$ composites. *Mater. Today Commun.* **25**, 101592 (2020).

11. Wang X, Gao X, Li M, Chen S, Sheng J, Yu J. Synthesis of flexible $BaTiO_3$ nanofibers for efficient vibration-driven piezocatalysis. *Ceram. Int.* **47**, 25416-25424 (2021).
12. Singh G, Sharma M, Vaish R. Flexible Ag@$LiNbO_3$/PVDF Composite Film for Piezocatalytic Dye/Pharmaceutical Degradation and Bacterial Disinfection. *ACS Appl. Mater. Interfaces* **13**, 22914-22925 (2021).
13. Wang J, *et al.* Energy and environmental catalysis driven by stress and temperature-variation. *J. Mater. Chem. A* **9**, 12400-12432 (2021).
14. Ganguli S, Hazra C, Chatti M, Samanta T, Mahalingam V. A Highly Efficient UV–Vis–NIR Active Ln^{3+}-Doped $BiPO_4$/$BiVO_4$ Nanocomposite for Photocatalysis Application. *Langmuir* **32**, 247-253 (2016).
15. Wang W, Li Y, Kang Z, Wang F, Yu JC. A NIR-driven photocatalyst based on α-$NaYF_4$:Yb,Tm@TiO_2 core–shell structure supported on reduced graphene oxide. *Appl. Catal., B* **182**, 184-192 (2016).
16. Tang Y, Di W, Zhai X, Yang R, Qin W. NIR-Responsive Photocatalytic Activity and Mechanism of $NaYF_4$:Yb,Tm@TiO_2 Core–Shell Nanoparticles. *ACS Catal.* **3**, 405-412 (2013).
17. Xu D-X, Lian Z-W, Fu M-L, Yuan B, Shi J-W, Cui H-J. Advanced near-infrared-driven photocatalyst: Fabrication, characterization, and photocatalytic performance of β-$NaYF_4$:Yb^{3+},Tm^{3+}@TiO_2 core@shell microcrystals. *Appl. Catal., B* **142-143**, 377-386 (2013).
18. Bai L, *et al.* Facet engineered interface design of $NaYF_4$:Yb,Tm upconversion nanocrystals on BiOCl nanoplates for enhanced near-infrared photocatalysis. *Nanoscale* **8**, 19014-19024 (2016).
19. Tou M, Mei Y, Bai S, Luo Z, Zhang Y, Li Z. Depositing CdS nanoclusters on carbon-modified $NaYF_4$:Yb,Tm upconversion nanocrystals for NIR-light enhanced photocatalysis. *Nanoscale* **8**, 553-562 (2016).
20. Tian Q, *et al.* Full-spectrum-activated Z-scheme photocatalysts based on $NaYF_4$:Yb^{3+}/Er^{3+}, TiO_2 and $Ag_6Si_2O_7$. *J. Mater. Chem. A* **5**, 23566-23576 (2017).

21. Huang S, *et al.* Near-infrared photocatalyst of Er^{3+}/Yb^{3+} codoped (CaF_2@TiO_2) nanoparticles with active-core/active-shell structure. *J. Mater. Chem. A* **1**, 7874-7879 (2013).
22. Guo X, Chen C, Zhang D, Tripp CP, Yin S, Qin W. Photocatalysis of $NaYF_4$:Yb,Er/CdSe composites under 1560 nm laser excitation. *RSC Adv.* **6**, 8127-8133 (2016).
23. Wang J, *et al.* Preparation of Bi_2S_3/carbon quantum dot hybrid materials with enhanced photocatalytic properties under ultraviolet-, visible- and near infrared-irradiation. *Nanoscale* **9**, 15873-15882 (2017).
24. Li Y, Cheng Z, Yao L, Yang S, Zhang Y. Boosting NIR-Driven Photocatalytic Activity of BiOBr:Yb^{3+}/Er^{3+}/Ho^{3+} Nanosheets by Enhanced Green Upconversion Emissions via Energy Transfer from Er^{3+} to Ho^{3+} Ions. *ACS Sustain. Chem. Eng.* **2019**, 18185–18196 (2019).
25. Lin YT, Lai SN, Wu JM. Simultaneous Piezoelectrocatalytic Hydrogen-Evolution and Degradation of Water Pollutants by Quartz Microrods@Few-Layered MoS_2 Hierarchical Heterostructures. *Adv. Mater.* **32**, 2002875 (2020).
26. Xu XL, *et al.* Harvesting vibration energy to piezo-catalytically generate hydrogen through Bi_2WO_6 layered-perovskite. *Nano Energy* **78**, 105351 (2020).
27. Wang MY, *et al.* Remarkably Enhanced Hydrogen Generation of Organolead Halide Perovskites via Piezocatalysis and Photocatalysis. *Adv. Energy Mater.* **9**, 1901801 (2019).
28. Sun YH, *et al.* Hydrogen Generation and Degradation of Organic Dyes by New Piezocatalytic 0.7$BiFeO_3$-0.3$BaTiO_3$ Nanoparticles with Proper Band Alignment. *ACS Appl. Mater. Interfaces* **13**, 11050-11057 (2021).
29. Yu C, *et al.* Ultrahigh piezocatalytic capability in eco-friendly $BaTiO_3$ nanosheets promoted by 2D morphology engineering. *J. Colloid Interface Sci* **596**, 288-296 (2021).
30. You HL, *et al.* Harvesting the Vibration Energy of $BiFeO_3$ Nanosheets for

Hydrogen Evolution. *Angew. Chem. Int. Ed.* **58**, 11779-11784 (2019).

31. Yang GD*, et al.* Cocatalyst Engineering in Piezocatalysis: A Promising Strategy for Boosting Hydrogen Evolution. *ACS Appl. Mater. Interfaces* **13**, 15305-15314 (2021).

32. Wang ZJ*, et al.* Enhanced H_2 Production of TiO_2/ZnO Nanowires Co-Using Solar and Mechanical Energy through Piezo-Photocatalytic Effect. *ACS Sustain. Chem. Eng.* **6**, 10162-10172 (2018).

33. Abbood HA, Alabadi A, Al-Hawash AB, Abbood AA, Huang KX. Square CdS Micro/Nanosheets as Efficient Photo/Piezo-bi-Catalyst for Hydrogen Production. *Catal. Lett.* **150**, 3059-3070 (2020).

34. Zhang MX, Zhao SY, Zhao ZC, Li S, Wang F. Piezocatalytic Effect Induced Hydrogen Production from Water over Non-noble Metal Ni Deposited Ultralong GaN Nanowires. *ACS Appl. Mater. Interfaces* **13**, 10916-10924 (2021).

35. Dai J*, et al.* Enhanced Piezocatalytic Activity of $Sr_{0.5}Ba_{0.5}Nb_2O_6$ Nanostructures by Engineering Surface Oxygen Vacancies and Self-Generated Heterojunctions. *ACS Appl. Mater. Interfaces* **13**, 7259-7267 (2021).
